# Post-COVID-19 social determinants of health and quality of life: a longitudinal study

**DOI:** 10.1590/1980-220X-REEUSP-2025-0565en

**Published:** 2026-06-12

**Authors:** Sabrina de Souza Gurgel Florencio, Francisca Elisângela Teixeira Lima, Glaubervania Alves Lima, Maria Gabriela Miranda Fontenele, Lívia Maia Pascoal, Lilia Jannet Saldarriaga Sandoval, Paulo César de Almeida, Gilvan Ferreira Felipe, Maria Williany Silva Ventura, Brena Shellem Bessa de Oliveira, Lorena Pinheiro Barbosa

**Affiliations:** 1 Universidade Federal do Ceará, Fortaleza, CE, Brazil.; 2 Universidade Federal do Maranhão, Imperatriz, MA, Brazil.; 3 Universidad Nacional de Tumbes, Tumbes, Peru.; 4 Universidade Estadual do Ceará, Fortaleza, CE, Brazil.; 5 Universidade da Integração Internacional da Lusofonia Afro-Brasileira, Redenção, CE, Brazil.

**Keywords:** COVID-19, Social Determinants of Health, Quality of Life, Nursing, Public Health

## Abstract

**Objective:**

To verify the association between the social determinants of health and quality of life domains in people in a post-COVID-19 condition.

**Methods:**

A longitudinal study was conducted with 179 people from the states of Ceará and Maranhão, followed for six months after a confirmed diagnosis of COVID-19. Data collection was performed using a link for COVID-19 patients monitoring software, sent monthly via phone messaging app. Variables related to the social determinants of health and the domains of the SF-36 questionnaire for assessing quality of life are considered. Data analyzed using descriptive and inferential statistics.

**Results:**

Women showed improvements in the pain domain between the 2nd and 4th month (p = 0.038), vitality between the 4th and 6th month (p = 0.035), and mental health between the 2nd and 6th month (p = 0.005). Men showed better recovery in overall health status at 2 and 4 months (p = 0.029) and in social aspects at 2 and 6 months (p = 0.040).

**Conclusion:**

Progressive improvement in quality of life was observed throughout the follow-up period, with differences between men and women in the areas of pain, vitality, mental health, general health status, and social aspects.

## INTRODUCTION

The COVID-19 pandemic triggered a global health crisis whose impacts extended beyond the acute phase of infection, generating prolonged repercussions on the physical, emotional, and social conditions of those affected, shaping the post-COVID-19 condition^(1,2)^. This scenario directly affected the quality of life (QoL) of the population, understood as an individual’s perception of their position in life, within the cultural context and values in which they are included^(3)^. During the pandemic, QoL became vulnerable to changes in daily habits, working conditions, housing, food, and access to health services^(4)^.

These elements comprise the Social Determinants of Health (SDH), which are social, economic, cultural, ethnic-racial, psychological, and behavioral factors influencing the risk of illness and mortality in populations^(5)^. To understand this interaction, this study adopts the Dahlgren and Whitehead model^(6)^, which organizes the SDHs into concentric layers, from individual factors, lifestyles, social and community networks, to living and working conditions and, finally, the socioeconomic macro-determinants. This structure highlights the complex interaction between the individual and their social and environmental context in determining health.

By acting on each of these layers, the COVID-19 pandemic functioned as a powerful intensifier of the structural inequalities already described in the Dahlgren and Whitehead model, making pre-existing vulnerabilities even more evident. In this context, groups with lower socioeconomic status, inadequate sanitation, precarious housing, and limited access to healthy food and health services were disproportionately affected^(7)^. These inequalities increased the risk of infection and mortality and compromised survivors’ quality of life. Although the literature has investigated post-COVID-19 QoL^(8,9)^, studies analyzing the influence of SDH on the evolution of QoL over time are still limited, especially in longitudinal investigations carried out in northeastern Brazilian states such as Ceará and Maranhão, which have historically been marked by more pronounced socioeconomic inequalities.

In Brazil, the pandemic highlighted historical gaps in access to basic social rights. The implementation of emergency measures, such as the Emergency Aid Program (*PAE*), a temporary financial benefit created by the federal government to reduce the economic impacts of the pandemic among socially vulnerable populations, while necessary, also exposed structural limitations in the State’s capacity for response and social protection^(10)^. In this scenario, evaluating the SDHs and their influence on post-COVID-19 QoL is fundamental to identifying the most impacted groups and supporting more equitable and effective public policies.

In this context, it becomes necessary to assess how the social determinants of health influence people’s quality of life after infection. The assessment of QoL, using instruments sensitive to temporal changes such as the *Short-Form Health Survey* (SF-36), becomes fundamental for identifying persistent vulnerabilities^(11)^. The longitudinal design constitutes a relevant methodological advantage, as it allows tracking the recovery trajectory over time, capturing variations in QoL domains and the influence of SDHs at different points in the post-COVID-19 period, something that cross-sectional studies fail to demonstrate. Therefore, it is necessary to understand which SDHs influence the QoL of people in a post-COVID-19 condition. This understanding contributes to the production of scientific knowledge and to the planning of health actions aimed at preventing illnesses, functional rehabilitation, and health promotion from a comprehensive and equitable perspective^(12)^.

Therefore, the present study aims to verify the association between the SDHs and QoL domains in people in a post-COVID-19 condition. The results can support nurses and health managers in developing longitudinal care strategies that consider regional inequities, guiding interventions aimed at functional rehabilitation, promoting mental health, and strengthening social support in the post-pandemic context.

## METHOD

### Design of Study

This is a longitudinal, analytical, and quantitative study conducted in the states of Ceará and Maranhão, regions that experienced a high incidence of COVID-19 during the pandemic. The follow-up took place over six months, with monthly submission of monitoring forms and application of the SF-36 in the 2nd, 4th, and 6th months, for individuals diagnosed with COVID-19.

### Study Protocol

The reference population consisted of 573,555 adults and older individuals (≥ 20 years old) with flu-like symptoms reported in the Influenza Surveillance Information System (*SIVEP-GRIPE*) and/or *e-SUS Notifica*, with 367,265 from Ceará and 206,290 from Maranhão. Considering a sampling error of 7% and a confidence interval of 95%, a minimum sample size of 179 participants was estimated.

The sample included individuals with a confirmed diagnosis of COVID-19 and a mobile phone number with internet access registered in the relevant information systems of the Health Departments of each state. Those with self-reported cognitive impairment or who did not respond after three attempts to contact them were excluded. Of the 391 eligible people who responded to the first form, there were 212 dropouts, mainly due to lack of response after further attempts to contact them (n = 168), refusal to continue participating (n = 28), and change or deactivation of the telephone number (n = 16), resulting in a final sample of 179 participants, 95 from Ceará and 84 from Maranhão.

For data collection, the software for COVID-19 patient monitoring was used, developed and validated in Brazil^(13)^, which contains two instruments: a sociodemographic and lifestyle characterization form, encompassing the SDHs (including variables of age, sex, race/color, physical activity, eating habits, sun exposure, sleep quality, smoking, alcohol consumption, household composition, cohabitation with at-risk groups, basic sanitation, and family income, organized according to the layers of the Dahlgren and Whitehead model); and the Short-Form Health Survey (SF-36), translated and validated for Brazil, consisting of 36 items distributed across eight domains (functional capacity, physical aspects, pain, general health status, vitality, social aspects, emotional aspects, and mental health)^(11)^.

Data collection took place between June 2021 and June 2022. Initially, data were obtained from the *SIVEP-GRIPE* and *e-SUS Notifica* systems, which record suspected and confirmed cases of COVID-19 in the country. The notification forms were completed by healthcare professionals and consolidated by the Epidemiological Surveillance units of the State Health Departments (*SESA*) of Ceará and Maranhão.

After authorization from *SESA*, data were exported to the monitoring software, which generated an individual link for each participant. This link, along with an invitation to participate, a Free Informed Consent Form (FICF), and a monthly follow-up form, was sent via WhatsApp, a messaging application widely used in Brazil. This may have favored the inclusion of participants with greater internet access and digital literacy, but it constitutes a limitation of the recruitment method. The SF-36 was administered at the second, fourth, and sixth months of follow-up. These intervals were defined based on literature on post-COVID-19 condition, which indicates the first six months as the period of greatest variability in clinical and functional recovery, with bimonthly assessments to capture progressive changes in QoL domains^(2,14)^. The deadline for a response was three days.

### Data Analysis

Data were analyzed using the software Statistical Package for the Social Sciences (SPSS), version 20.0, license 10101131007. The variables were described using absolute and relative frequencies, means, and standard deviations. The normality of the data was assessed using the Kolmogorov-Smirnov test.

Given the non-normal distribution, the Friedman test was applied, followed by the Conover test for multiple comparisons between the 2nd, 4th, and 6th months, aiming to verify the evolution of the SF-36 domains and their association with the social determinants of health. For comparisons between independent groups, the Kruskal-Wallis and Mann-Whitney tests were used. For variables with significant differences according to the Kruskal-Wallis test, a Dunn post-hoc test with Bonferroni correction was performed. A significance level of p < 0.05 was adopted.

### Ethical Aspects

The study was approved by the Research Ethics Committee, under opinion number 4.278.495/2020 and identifier CAAE 36193820.1.0000.5054.

## RESULTS

The study included 179 individuals, predominantly women (58.7%), with a mean age of 37.4 ± 12.6 years and self-identifying as mixed race (56.4%). Low adherence to regular physical activity (81.0%), insufficient consumption of fruits and vegetables (52.5%), high intake of fried and processed foods (73.2%), low sun exposure (60.9%), and difficulty sleeping (55.3%) were observed. Alcohol consumption was reported by 44.1%, and 4.5% were smokers. Most lived with two or more people (95.0%), lived with individuals from the at-risk group (50.3%), had access to the general water distribution network (93.9%), sewage network (67.0%) and lived with a monthly income of up to three minimum wages (59.8%) (Table 1).

**Table 1:** Association of social determinants of health in people with COVID-19 with the averages of the functional capacity and physical limitations domains of the SF-36 instrument, comparing the 2nd, 4th and 6th month – Fortaleza, CE, Brazil, 2023.

SF-36 domains	Total			Functional capacity average (±SD)					Limitations due to physical aspects average (±SD)			
	n	%		2nd month	4th month	6th month	p*		2nd month	4th month	6th month	p*
**Layer 1 – Age, sex, and hereditary factors**												
**Age**												
20–39	114	63.7		83.4 (±20.4)	83.7 (±19.2)	84.2 (±18.4)	0.905		76.5 (±35.7)	82.2 (±30.9)	84.2 (±27.6)	0.070
40–59	56	31.3		75.8 (±22.8)	76.9 (±23.6)	78.8 (±21.2)	**0.038**		71.4 (±37.9)	73.2 (±38.6)	74.5 (±36.1)	0.645
60–75	9	5.0		66.6 (±25.1)	68.3 (±23.3)	69.4 (±16.2)	0.747		66.6 (±45.0)	75.0 (±39.5)	69.4 (±46.3)	0.819
**p-value** ^§^				**0.008**	**0.034**	**0.027**			0.634	0.432	0.254	
**Sex**												
Male	105	58.7		87.4 (±17.0)	86.6 (±18.5)	88.1 (±17.1)	0.591		80.0 (±34.8)	88.5 (±25.5)	89.8 (±23.0)	**0.038**
Female	74	41.3		75.1 (±23.4)	76.6 (±22.1)	77.4 (±19.9)	0.331		70.4 (±37.7)	72.3 (±37.6)	73.8 (±35.4)	0.467
**p-value** ^†^				**<0.001**	**0.001**	**<0.001**			**0.042**	**0.002**	**0.001**	
**Race/Color**												
White	57	31.8		79.3 (±26.4)	80.5 (±22.2)	83.0 (±20.2)	0.560		74.5 (±39.1)	76.3 (±33.8)	77.6 (±32.2)	0.672
Black	21	11.7		80.0 (±21.8)	78.8 (±23.6)	76.1 (±20.9)	0.115		71.4 (±35.6)	70.2 (±40.7)	80.9 (±34.3)	0.353
Brown	101	56.4		80.7 (±18.9)	81.4 (±20.3)	82.3 (±18.7)	0.383		75.0 (±36.0)	82.4 (±32.5)	81.9 (±31.2)	**0.024**
**p-value** ^§^				0.745	0.951	0.307			0.754	0.208	0.549	
**2nd layer – Individuals’ lifestyle**												
**Physical activity/week**												
0 to 3 days	145	81.0		78.6 (±21.9)	79.2 (±21.6)	80.5 (±19.7)	0.236		71.5 (±37.8)	79.3 (±34.1)	81.5 (±30.7)	**<0.001**
4 to 7 days	34	19.0		86.9 (±20.1)	87.9 (±18.3)	87.3 (±17.7)	0.500		86.7 (±29.6)	77.9 (±34.1)	75.7 (±36.1)	**0.026**
**p-value** ^†^				**0.010**	**0.010**	**0.042**			**0.009**	0.832	0.583	
**Fruit and vegetable consumption/week**												
0 to 3 days	94	52.5		78.3 (±21.6)	79.8 (±20.8)	80.7 (±19.7)	0.179		76.5 (±34.5)	78.9 (±32.4)	81.1 (±29.9)	0.300
4 to 7 days	85	47.5		82.2 (±21.9)	81.9 (±21.7)	83.0 (±19.2)	0.805		72.0 (±39.2)	79.1 (±35.9)	79.7 (±33.9)	0.121
**p-value** ^†^				0.071	0.280	0.391			0.760	0.686	0.810	
**Consumption of fried and processed foods/week**												
0 to 3 days	131	73.2		79.3 (±22.5)	81.1 (±21.9)	83.0 (±19.2)	**0.026**		75.0 (±35.7)	79.0 (±33.9)	79.9 (±31.5)	0.082
4 to 7 days	48	26.8		82.5 (±19.5)	80.1 (±19.3)	78.6 (±20.1)	0.136		72.9 (±39.8)	79.1 (±34.7)	81.7 (±32.9)	0.353
**p-value** ^†^				0.420	0.912	0.119			0.818	0.482	0.510	
**Sun exposure/week**												
0 to 3 days	109	60.9		79.0 (±22.0)	82.1 (±19.5)	82.1 (±18.9)	0.110		78.8 (±32.6)	81.6 (±31.1)	81.8 (±30.2)	0.202
4 to 7 days	70	39.1		82.0 (±21.4)	78.8 (±23.6)	81.3 (±20.4)	0.580		67.5 (±41.7)	75.0 (±38.0)	78.2 (±34.2)	0.198
**p-value** ^†^				0.254	0.636	0.922			0.147	0.334	0.589	
**Difficulty sleeping**												
Yes	99	55.3		77.4 (±22.8)	80.5 (±21.4)	79.9 (±20.7)	0.170		82.1 (±31.3)	85.0 (±29.9)	85.9 (±26.3)	0.133
No	80	44.7		83.6 (±20.0)	81.3 (±21.0)	84.1 (±17.7)	0.591		68.1 (±39.7)	74.2 (±36.5)	76.0 (±35.1)	0.340
**p-value** ^†^				**0.035**	0.748	0.244			**0.019**	**0.022**	0.059	
**Smoking**												
Yes	8	4.5		86.2 (±24.0)	90.6 (±10.5)	87.5 (±18.5)	0.953		100 (±00.0)	90.6 (±26.5)	100 (±00.0)	0.368
No	171	95.5		79.9 (±21.7)	80.4 (±21.5)	81.5 (±19.5)	0.427		73.2 (±37.2)	78.5 (±34.3)	79.5 (±32.2)	**0.041**
**p-value** ^†^				0.167	0.319	0.337			**0.019**	0.224	**0.043**	
**Alcoholism**												
Yes	79	44.1		83.1 (±20.3)	83.2 (±19.9)	84.3 (±18.0)	0.756		76.8 (±35.0)	82.2 (±30.7)	85.1 (±27.8)	0.164
No	100	55.9		77.9 (±22.7)	79.0 (±22.1)	79.9 (±20.4)	0.160		72.5 (±38.1)	76.5 (±36.3)	76.7 (±34.3)	0.256
**p-value** ^†^				0.136	0.274	0.211			0.461	0.388	0.126	
**3rd layer – Social and community networks**												
**Number of people residing in the household**												
Lives alone or one	9	5.0		75.5 (±23.7)	76.1 (±25.0)	78.8 (±26.1)	0.857		72.2 (±42.2)	72.2 (±44.0)	91.6 (±17.6)	0.779
≥ 2	170	95.0		80.4 (±21.7)	81.1 (±21.0)	82.0 (±19.1)	0.481		74.5 (±36.6)	79.4 (±33.5)	79.8 (±32.3)	**0.043**
**p-value** ^†^				0.471	0.551	0.970			0.819	0.802	0.351	
**Lives with people in the at-risk group**												
Yes	90	50.3		79.4 (±21.6)	80.7 (±20.7)	82.3 (±20.0)	0.819		73.3 (±37.8)	80.2 (±33.6)	80.8 (±32.4)	**0.018**
No	89	49.7		80.9 (±22.0)	80.9 (±21.8)	81.3 (±19.0)	0.114		75.5 (±35.9)	77.8 (±34.6)	80.0 (±31.3)	0.416
**p-value** ^†^				0.525	0.678	0.717			0.687	0.597	0.815	
**4th layer – Living conditions**												
**Water supply**												
Distribution network	168	93.9		81.1 (±20.2)	81.5 (±21.0)	82.3 (±19.1)	0.387		74.1 (±36.9)	80.0 (±33.5)	81.5 (±30.4)	**0.017**
Other	11	6.1		66.8 (±37.7)	70.4 (±22.6)	74.0 (±24.5)	0.882		79.5 (±35.0)	63.6 (±39.3)	63.6 (±46.5)	0.444
**p-value** ^†^				0.508	0.054	0.276			0.642	0.119	0.212	
**Sewer drain**												
Sewer system	120	67.0		79.7 (±20.7)	81.5 (±19.6)	81.5 (±19.3)	0.526		73.9 (±36.8)	80.6 (±33.2)	81.6 (±29.6)	**0.042**
Other	59	33.0		81.2 (±24.0)	79.5 (±24.2)	82.5 (±19.9)	0.853		75.4 (±36.9)	75.8 (±35.6)	77.9 (±35.9)	0.764
**p-value** ^†^				0.293	0.861	0.465			0.537	0.446	0.922	
**5th layer – General socioeconomic, cultural and environmental conditions**												
**Family income**												
< 3 minimum wages	107	59.8		78.8 (±22.3)	79.5 (±21.4)	79.7 (±20.4)	0.598		73.5 (±37.7)	80.3 (±32.5)	81.3 (±31.6)	**0.027**
>3 minimum wages	72	40.2		82.2 (±20.9)	82.8 (±20.9)	85.0 (±17.6)	0.532		75.6 (±35.5)	77.0 (±36.2)	79.1 (±32.2)	0.856
**p-value** ^†^				0.311	0.299	0.142			0.843	0.711	0.510	

Legend: *Friedman Test/

^§^Kruskal-Wallis/

^†^Mann-Whitney.

Longitudinal analysis of the association between the SDHs and the SF-36 domains revealed significant variations across the 2nd, 4th, and 6th months of follow-up. These differences occurred according to sociodemographic characteristics, lifestyles, environmental conditions, and social networks (Tables 1, 2, and 3).

**Table 2:** Association of social determinants of health in people with COVID-19 with the averages of the pain, general health status and vitality domains of the SF-36 instrument, comparing the 2nd, 4th and 6th month – Fortaleza, CE, Brazil, 2023.

SF-36 domains	Pain average (±SD)				General health status average (±SD)				Vitality average (±SD)			
	2nd month	4th month	6th month	p*	2nd month	4th month	6th month	p*	2nd month	4th month	6th month	p*
**Layer 1 – Age, sex, and hereditary factors**												
**Age**												
20–39	72.5 (±23.1)	73.5 (±22.4)	74.4 (±22.4)	0.514	64.4 (±20.5)	65.5 (±20.6)	66.4 (±21.2)	0.507	53.7 (±18.9)	54.3 (±22.0)	56.4 (±19.9)	0.246
40–59	66.4 (±24.4)	70.5 (±23.2)	72.0 (±23.6)	0.015	64.0 (±17.3)	68.7 (±18.3)	66.1 (±19.2)	0.096	56.8 (±19.4)	60.0 (±19.4)	62.2 (±19.0)	**0.006**
60–75	60.3 (±18.3)	65.1 (±27.5)	53.0 (±11.0)	0.485	52.3 (±8.9)	54.7 (±10.9)	55.3 (±12.2)	0.678	52.2 (±12.0)	53.8 (±13.8)	56.6 (±13.2)	0.482
**p-value** ^§^	0.155	0.555	**0.016**		0.090	0.074	0.152		0.563	0.278	0.217	
**Sex**												
Male	76.3 (±22.5)	75.8 (±22.4)	78.0 (±21.4)	0.555	68.2 (±17.6)	72.0 (±18.9)	71.1 (±18.3)	**0.045**	61.4 (±19.3)	64.3 (±20.2)	63.9 (±18.6)	0.161
Female	65.6 (±23.2)	69.5 (±23.0)	68.8 (±23.1)	**0.035**	60.5 (±19.8)	61.7 (±19.2)	62.0 (±20.9)	0.839	49.8 (±16.9)	50.3 (±19.6)	54.3 (±19.1)	**0.009**
**p-value** ^†^	**0.003**	0.068	**0.009**		**0.013**	**<0.001**	**0.004**		**<0.001**	**<0.001**	**0.001**	
**Race/Color**												
White	64.1 (±26.2)	69.6 (±24.4)	70.9 (±24.5)	0.036	62.2 (±22.8)	64.2 (±23.2)	63.2 (±22.4)	0.064	53.7 (±21.1)	54.0 (±22.4)	53.5 (±20.5)	0.856
Black	72.5 (±22.4)	65.5 (±24.0)	75.3 (±21.5)	0.220	62.2 (±16.3)	63.8 (±19.2)	62.2 (±12.7)	0.334	55.0 (±18.5)	52.1 (±23.6)	53.8 (±18.9)	0.776
Brown	72.8 (±21.6)	74.9 (±21.6)	73.0 (±22.1)	0.073	64.8 (±17.6)	67.4 (±17.6)	67.9 (±20.2)	0.035	55.0 (±17.6)	58.1 (±19.5)	61.8 (±18.3)	**<0.001**
**p-value** ^§^	0.131	0.199	0.825		0.771	0.603	0.161		0.925	0.402	**0.024**	
**2nd layer – Individuals’ lifestyle**												
**Physical activity/week**												
0 to 3 days	68.2 (±23.3)	70.2 (±22.7)	71.7 (±23.0)	**0.048**	62.8 (±18.9)	64.6 (±19.3)	64.7 (±19.8)	0.215	53.3 (±19.0)	54.8 (±21.5)	57.6 (±19.7)	**0.015**
4 to 7 days	77.7 (±22.7)	80.1 (±22.3)	76.3 (±21.8)	0.530	67.4 (±20.7)	72.1 (±20.4)	70.1 (±21.9)	0.512	60.1 (±17.1)	61.4 (±18.0)	60.8 (±18.4)	0.539
**p-value** ^†^	**0.028**	**0.021**	0.312		0.257	0.059	0.159		0.050	0.125	0.402	
**Fruit and vegetable consumption/week**												
0 to 3 days	69.9 (±23.0)	71.6 (±21.9)	72.1 (±22.8)	0.174	61.1 (±20.0)	64.4 (±19.8)	62.9 (±20.7)	0.215	54.2 (±18.2)	55.7 (±21.8)	58.2 (±19.7)	0.056
4 to 7 days	70.2 (±24.1)	72.6 (±24.0)	73.1 (±22.9)	0.208	66.5 (±18.1)	67.8 (±19.5)	68.8 (±19.5)	0.589	55.1 (±19.4)	56.4 (±20.1)	58.2 (±19.3)	0.150
**p-value** ^†^	0.831	0.610	0.699		0.084	0.278	0.069		0.754	0.871	0.853	
**Consumption of fried and processed foods/week**												
0 to 3 days	70.4 (±23.4)	72.9 (±23.0)	73.4 (±22.2)	0.168	64.4 (±19.1)	67.2 (±18.5)	67.3 (±19.7)	0.235	55.1 (±18.4)	56.6 (±21.0)	59.7 (±19.0)	**0.006**
4 to 7 days	69.1 (±23.7)	69.8 (±22.6)	70.5 (±24.3)	0.184	61.7 (±19.7)	62.7 (±22.4)	61.3 (±21.4)	0.344	53.2 (±19.8)	54.5 (±21.0)	54.2 (±20.2)	0.779
**p-value** ^†^	0.754	0.278	0.557		0.548	0.539	0.097		0.500	0.397	0.139	
**Sun exposure/week**												
0 to 3 days	71.2 (±23.7)	73.1 (±22.6)	73.3 (±22.7)	0.168	63.1 (±19.1)	66.4 (±19.1)	65.8 (±20.2)	**0.041**	54.8 (±17.7)	57.4 (±21.0)	60.0 (±18.1)	**0.007**
4 to 7 days	68.1 (±23.2)	70.6 (±23.4)	71.6 (±22.9)	0.214	64.6 (±19.5)	65.4 (±20.7)	65.6 (±20.5)	0.983	54.4 (±20.5)	54.0 (±20.9)	55.6 (±21.2)	0.593
**p-value** ^†^	0.323	0.509	0.670		0.501	0.896	0.895		0.999	0.340	0.200	
**Difficulty sleeping**												
Yes	66.8 (±24.5)	69.4 (±23.1)	70.1 (±23.0)	**0.011**	60.4 (±18.5)	63.6 (±20.1)	64.2 (±20.6)	**0.039**	51.3 (±19.6)	53.7 (±21.5)	54.7 (±20.4)	0.127
No	74.0 (±21.7)	75.5 (±22.3)	75.6 (±22.2)	0.860	67.8 (±19.5)	68.9 (±18.9)	67.6 (±19.8)	0.279	58.8 (±16.9)	59.0 (±20.1)	62.6 (±17.3)	0.053
**p-value** ^†^	**0.045**	0.099	0.118		**0.010**	0.074	0.368		**0.008**	0.060	**0.010**	
**Smoking**												
Yes	79.7 (±24.0)	67.5 (±24.9)	79.6 (±16.2)	0.228	69.8 (±20.1)	62.3 (±15.4)	63.3 (±26.0)	0.058	60.0 (±17.5)	61.8 (±17.5)	68.1 (±16.4)	**0.040**
No	69.6 (±23.4)	72.3 (±22.9)	72.3 (±22.0)	**0.014**	63.4 (±19.2)	66.2 (±19.9)	65.8 (±20.1)	0.326	54.4 (±18.8)	55.8 (±21.1)	57.8 (±19.5)	**0.035**
**p-value** ^†^	0.243	0.481	0.455		0.452	0.561	0.740		0.408	0.449	0.169	
**Alcoholism**												
Yes	73.8 (±23.5)	72.6 (±22.7)	75.3 (±21.1)	0.618	66.2 (±19.5)	66.7 (±19.3)	69.1 (±18.2)	0.377	56.0 (±18.6)	55.6 (±21.5)	59.3 (±20.4)	0.374
No	67.0 (±23.1)	71.7 (±23.2)	70.4 (±23.9)	**0.008**	61.7 (±18.9)	65.4 (±20.0)	63.1 (±21.5)	0.091	53.5 (±18.9)	58.5 (±20.6)	57.4 (±18.7)	**0.012**
**p-value** ^†^	0.060	0.801	0.216		0.125	0.826	0.092		0.376	0.903	0.414	
**3rd layer – Social and community networks**												
**Number of people residing in the household**												
Lives alone or one	63.0 (±22.6)	69.0 (±22.8)	72.5 (±24.9)	0.152	59.8 (±19.0)	63.4 (±20.0)	61.8 (±27.7)	0.401	53.3 (±16.0)	54.4 (±20.6)	65.0 (±22.0)	0.087
≥ 2	70.4 (±23.5)	72.3 (±22.8)	72.6 (±22.7)	0.091	63.9 (±19.3)	66.1 (±19.7)	65.9 (±19.9)	0.169	54.7 (±18.9)	56.2 (±21.1)	57.9 (±19.3)	**0.025**
**p-value** ^†^	0.335	0.705	0.973		0.335	0.501	0.756		0.804	0.796	0.255	
**Lives with people in the at-risk group**												
Yes	70.1 (±22.5)	72.2 (±22.1)	73.1 (±22.3)	0.273	64.6 (±20.1)	65.4 (±19.5)	67.3 (±19.8)	0.131	55.0 (±18.4)	53.7 (±21.2)	58.8 (±19.2)	**0.026**
No	69.9 (±24.5)	72.0 (±23.8)	72.1 (±23.3)	0.115	62.7 (±18.4)	66.5 (±19.9)	64.1 (±20.8)	**0.026**	54.3 (±19.3)	58.4 (±20.7)	57.6 (±19.7)	**0.011**
**p-value** ^†^	0.913	0.984	0.781		0.461	0.648	0.359		0.729	0.271	0.791	
**4th layer – Living conditions**												
Water supply												
Distribution network	70.1 (±23.2)	71.8 (±22.9)	72.6 (±22.9)	0.062	63.6 (±19.3)	66.1 (±19.8)	65.7 (±20.4)	0.109	54.8 (±18.9)	56.6 (±21.0)	58.7 (±19.5)	**0.009**
Other	68.1 (±27.7)	76.7 (±23.2)	77.2 (±20.9)	0.422	65.2 (±19.5)	64.0 (±18.2)	66.6 (±18.8)	1.000	51.3 (±17.3)	48.6 (±20.5)	50.9 (±17.8)	0.773
**p-value** ^†^	0.887	0.450	0.966		0.675	0.711	0.873		0.613	0.233	0.204	
**Sewer drain**												
Sewer system	68.6 (±23.5)	71.6 (±23.0)	71.7 (±23.6)	**0.045**	63.6 (±19.2)	65.7 (±20.3)	65.6 (±20.1)	0.182	53.3 (±18.1)	53.7 (±19.8)	56.9 (±18.5)	**0.017**
Other	72.9 (±23.2)	73.2 (±22.9)	74.5 (±21.0)	0.622	63.8 (±19.4)	66.7 (±18.5)	66.1 (±20.9)	0.640	57.3 (±20.0)	61.0 (±22.6)	61.1 (±21.1)	0.302
**p-value** ^†^	0.216	0.623	0.434		0.881	0.746	0.866		0.155	**0.023**	0.195	
**5th layer – General socioeconomic, cultural and environmental conditions**												
**Family income**												
< 3 minimum wages	70.8 (±23.6)	73.9 (±22.8)	73.4 (±23.4)	0.150	63.4 (±18.8)	65.1 (±19.5)	63.8 (±20.4)	0.468	54.8 (±19.6)	57.0 (±21.3)	57.7 (±20.1)	**0.048**
>3 minimum wages	68.9 (±23.4)	69.4 (±23.0)	71.4 (±22.0)	0.109	64.0 (±20.1)	67.3 (±19.9)	68.5 (±20.0)	0.130	54.4 (±17.5)	54.7 (±20.5)	59.0 (±18.5)	**0.034**
**p-value** ^†^	0.593	0.197	0.455		0.664	0.433	0.123		0.972	0.473	0.656	

Legend: *Friedman Test/

^§^Kruskal-Wallis/

^†^Mann-Whitney.

**Table 3:** Association of social determinants of health in people with COVID-19 with the averages of the social aspects, emotional aspects and mental health domains of the SF-36 instrument, comparing the 2nd, 4th and 6th month – Fortaleza, CE, Brazil, 2023.

SF-36 domains	Social aspects average (±SD)					Emotional aspects average (±SD)					Mental health average (±SD)			
	2nd month	4th month	6th month	p*		2nd month	4th month	6th month	p*		2nd month	4th month	6th month	p*
**Layer 1 – Age, sex, and hereditary factors**														
**Age**														
20–39	70.5 (±24.7)	73.3 (±24.8)	72.5 (±24.4)	0.220		63.4 (±40.1)	71.6 (±37.1)	70.7 (±38.4)	**0.025**		60.3 (±18.6)	63.5 (±19.3)	65.5 (±20.3)	**0.013**
40–59	72.3 (±23.9)	77.0 (±25.4)	79.0 (±23.2)	**0.002**		76.7 (±37.0)	74.4 (±40.1)	75.5 (±38.9)	0.705		64.0 (±19.7)	65.2 (±21.5)	67.5 (±19.5)	0.204
60–75	63.8 (±20.1)	69.4 (±26.5)	79.1 (±22.5)	0.125		59.2 (±49.3)	62.9 (±48.4)	77.7 (±44.0)	0.368		53.3 (±12.6)	53.7 (±15.1)	60.4 (±15.9)	**0.042**
**p-value** ^§^	0.508	0.479	0.212			0.056	0.596	0.537			0.163	0.240	0.482	
**Sex**														
Male	77.5 (±22.5)	80.9 (±20.5)	81.9 (±21.4)	**0.014**		76.5 (±34.7)	82.8 (±30.8)	83.7 (±31.8)	0.179		68.3 (±17.6)	70.9 (±19.5)	72.3 (±18.5)	0.101
Female	65.9 (±24.3)	69.6 (±26.9)	70.0 (±24.7)	0.061		60.9 (±42.2)	64.4 (±41.6)	64.7 (±41.3)	0.514		56.1 (±12.6)	58.4 (±18.6)	61.3 (±19.4)	0.009
**p-value** ^†^	**0.001**	**0.006**	**0.002**			**0.009**	**0.003**	**0.001**			**<0.001**	**<0.001**	**<0.001**	
**Race/Color**														
White	65.1 (±28.5)	71.9 (±26.5)	72.1 (±23.9)	**0.004**		64.9 (±41.0)	69.5 (±36.3)	71.3 (±38.0)	0.686		57.6 (±20.2)	61.8 (±20.4)	62.8 (±21.0)	**0.031**
Black	72.0 (±26.4)	69.0 (±26.6)	72.0 (±28.4)	0.932		71.4 (±39.8)	61.9 (±43.8)	66.6 (±44.7)	0.907		62.8 (±20.0)	63.0 (±20.6)	64.1 (±18.8)	0.914
Brown	73.6 (±20.6)	76.7 (±23.7)	77.1 (±23.1)	0.065		67.9 (±39.6)	75.5 (±38.5)	74.5 (±38.0)	0.067		62.8 (±17.5)	64.7 (±19.6)	67.9 (±19.3)	**0.003**
**p-value** ^§^	0.283	0.340	0.423			0.707	0.144	0.781			0.317	0.662	0.354	
**2nd layer – Individuals’ lifestyle**														
**Physical activity/week**														
0 to 3 days	70.5 (±24.8)	73.1 (±25.1)	75.0 (±24.4)	**0.015**		66.2 (±40.2)	71.9 (±38.6)	73.7 (±38.1)	**0.036**		60.6 (±19.8)	62.8 (±20.6)	65.6 (±19.9)	**0.003**
4 to 7 days	71.6 (±22.0)	79.4 (±24.4)	74.6 (±22.9)	**0.016**		72.5 (±38.9)	72.5 (±38.8)	67.6 (±41.4)	0.617		63.2 (±13.7)	66.9 (±16.5)	66.8 (±19.7)	0.417
**p-value** ^†^	0.952	0.144	0.844			0.328	0.957	0.415			0.452	0.249	0.721	
**Fruit and vegetable consumption/week**														
0 to 3 days	73.2 (±23.7)	75.5 (±23.3)	76.7 (±23.3)	0.062		70.2 (±38.3)	71.6 (±38.4)	77.3 (±34.9)	0.094		60.7 (±19.2)	63.5 (±20.7)	66.0 (±19.7)	**0.014**
4 to 7 days	67.9 (±24.6)	72.9 (±26.9)	72.9 (±24.9)	**0.018**		64.3 (±41.7)	72.5 (±38.8)	76.4 (±42.0)	0.118		61.6 (±18.4)	63.6 (±19.1)	65.7 (±20.0)	0.075
**p-value** ^†^	0.131	0.658	0.299			0.443	0.800	0.152			0.731	0.964	0.986	
**Consumption of fried and processed foods/week**														
0 to 3 days	71.4 (±23.6)	75.4 (±24.2)	77.3 (±23.0)	**<0.001**		67.6 (±39.8)	71.2 (±38.7)	75.8 (±37.4)	0.073		61.3 (±17.9)	63.7 (±20.0)	66.7 (±19.5)	**0.001**
4 to 7 days	68.7 (±25.9)	71.0 (±27.1)	68.2 (±25.7)	0.239		66.6 (±40.6)	74.3 (±38.4)	63.8 (±41.1)	**0.032**		60.6 (±21.1)	63.2 (±19.7)	63.5 (±20.6)	0.340
**p-value** ^†^	0.582	0.573	**0.025**			0.870	0.573	0.057			0.900	0.860	0.423	
**Sun exposure/week**														
0 to 3 days	72.9 (±23.1)	77.2 (±23.3)	78.4 (±23.1)	**0.003**		71.5 (±37.6)	75.8 (±37.6)	77.6 (±34.8)	0.180		61.1 (±18.4)	63.9 (±20.1)	68.0 (±18.4)	**0.002**
4 to 7 days	67.3 (±25.7)	69.6 (±26.9)	69.4 (±24.6)	0.212		60.9 (±42.8)	66.1 (±39.5)	64.7 (±43.1)	0.531		61.1 (±19.5)	63.0 (±19.6)	62.6 (±21.5)	0.312
**p-value** ^†^	0.164	0.061	**0.010**			0.134	0.050	0.069			0.841	0.798	0.140	
**Difficulty sleeping**														
Yes	65.0 (±25.5)	69.9 (±25.3)	71.3 (±24.4)	**0.004**		59.5 (±41.8)	63.9 (±41.6)	66.3 (±42.4)	0.290		55.5 (±19.0)	59.4 (±20.7)	62.1 (±20.9)	**<0.001**
No	77.8 (±20.5)	79.6 (±23.8)	79.3 (±23.0)	0.239		77.0 (±35.4)	82.0 (±31.7)	80.4 (±32.1)	0.345		68.1 (±16.0)	68.7 (±17.7)	70.6 (±17.4)	0.575
**p-value** ^†^	**0.001**	**0.006**	**0.016**			**0.004**	**0.002**	**0.038**			**<0.001**	**0.002**	**0.005**	
Smoking														
Yes	78.1 (±19.7)	76.5 (±24.4)	79.6 (±25.8)	0.846		91.6 (±15.4)	87.5 (±17.2)	87.5 (±24.8)	0.819		61.5 (±11.6)	64.5 (±20.1)	67.5 (±18.3)	0.670
No	70.3 (±24.4)	74.1 (±25.1)	74.7 (±24.0)	**0.001**		66.2 (±40.4)	71.3 (±39.1)	71.9 (±39.1)	0.082		61.1 (±19.1)	63.5 (±19.9)	65.8 (±19.9)	**0.002**
**p-value** ^†^	0.449	0.808	0.533			0.104	0.455	0.313			0.964	0.958	0.911	
Alcoholism														
Yes	73.8 (±23.2)	75.0 (±25.7)	78.3 (±23.4)	0.117		68.7 (±39.7)	75.5 (±37.6)	77.2 (±36.8)	0.069		61.8 (±16.7)	63.8 (±18.8)	67.0 (±19.3)	0.096
No	68.2 (±24.8)	73.7 (±24.5)	72.2 (±24.3)	**0.005**		66.3 (±40.3)	69.3 (±39.2)	69.0 (±39.9)	0.707		60.6 (±20.3)	63.4 (±20.8)	65.0 (±20.2)	**0.011**
**p-value** ^†^	0.129	0.639	0.090			0.702	0.261	0.156			0.836	0.899	0.521	
**3rd layer – Social and community networks**														
**Number of people residing in the household**														
Lives alone or one	79.1 (±15.3)	61.1 (±26.1)	72.2 (±23.1)	0.355		59.2 (±49.3)	44.4 (±44.0)	85.1 (±29.3)	0.115		58.2 (±19.7)	62.6 (±23.1)	71.5 (±19.1)	**0.008**
≥ 2	70.2 (±24.5)	75.0 (±24.8)	75.0 (±24.1)	**<0.001**		67.8 (±39.5)	73.5 (±37.8)	71.9 (±39.1)	0.116		61.3 (±18.8)	63.6 (±19.8)	65.6 (±19.8)	**0.008**
**p-value** ^†^	0.391	0.111	0.597			0.718	**0.048**	0.288			0.506	0.869	0.391	
**Lives with people in the at-risk group**														
Yes	70.8 (±24.2)	75.4 (±24.2)	76.3 (±22.6)	**0.005**		67.4 (±40.8)	71.8 (±38.0)	74.4 (±39.3)	0.160		60.5 (±17.6)	61.1 (±19.1)	64.7 (±18.4)	0.131
No	70.6 (±24.4)	73.1 (±25.9)	73.4 (±25.4)	0.158		67.4 (±39.2)	72.2 (±39.3)	70.7 (±38.2)	0.397		61.7 (±19.9)	66.1 (±20.5)	67.1 (±21.2)	**0.004**
**p-value** ^†^	0.973	0.698	0.608			0.898	0.761	0.394			0.742	0.132	0.295	
**4th layer – Living conditions**														
**Water supply**														
Distribution network	70.6 (±24.4)	74.5 (±25.2)	75.6 (±23.9)	**<0.001**		67.6 (±39.8)	72.8 (±38.1)	74.2 (±38.0)	0.081		61.1 (±19.0)	64.1 (±19.8)	66.2 (±19.9)	**<0.001**
Other	71.5 (±21.7)	70.4 (±23.2)	63.6 (±24.6)	0.200		63.6 (±43.3)	60.6 (±44.2)	48.4 (±43.1)	0.174		61.8 (±15.4)	54.9 (±19.1)	60.3 (±17.9)	0.417
**p-value** ^†^	0.976	0.445	0.083			0.672	0.315	**0.020**			0.969	0.174	0.282	
**Sewer drain**														
Sewer system	69.4 (±24.0)	73.1 (±24.7)	75.2 (±23.2)	**0.003**		65.8 (±39.4)	72.2 (±38.7)	73.0 (±37.2)	0.103		59.8 (±18.6)	61.4 (±19.1)	65.4 (±19.0)	**0.002**
Other	73.3 (±24.6)	76.6 (±25.6)	74.3 (±25.8)	0.190		70.6 (±41.0)	71.7 (±38.5)	71.7 (±41.8)	0.837		63.7 (±19.0)	68.0 (±20.9)	66.7 (±21.5)	0.100
**p-value** ^†^	0.269	0.241	0.995			0.239	0.766	0.910			0.221	**0.029**	0.652	
**5th Layer – General socioeconomic, cultural and environmental conditions**														
**Family income**														
< 3 minimum wages	70.5 (±24.4)	74.2 (±25.2)	73.9 (±25.1)	**0.007**		69.1 (±39.3)	72.8 (±37.7)	73.2 (±38.9)	0.293		61.2 (±19.8)	64.1 (±20.4)	65.3 (±21.0)	**0.040**
>3 minimum wages	71.0 (±24.1)	74.3 (±24.9)	76.3 (±22.4)	0.117		64.8 (±41.0)	70.8 (±39.9)	71.7 (±38.6)	0.404		61.0 (±17.2)	62.7 (±19.1)	66.7 (±17.9)	**0.025**
**p-value** ^†^	0.918	0.939	0.718			0.551	0.818	0.637			0.984	0.637	0.776	

Legend: *Friedman Test/

^§^Kruskal-Wallis/

^†^Mann-Whitney.

In the first layer, participants aged 40 to 59 years showed significant improvement between the 2nd and 6th month in functional capacity (p = 0.033), pain (p = 0.042), vitality (p = 0.009), and social aspects (p = 0.012). Among young adults (20 to 39 years old) and older adults (60 to 75 years old), the improvement was concentrated in mental health, with the greatest variation between the 2nd and 6th month (p = 0.009 and p = 0.045, respectively). Comparatively, participants aged 20 to 39 years maintained higher functional capacity scores than those aged 40 to 59 years and 60 to 75 years in the 2nd month (p = 0.016 and p = 0.021, respectively), sustaining this difference over the older people in the 4th (p = 0.026) and 6th months (p = 0.013). In the 6th month, age also influenced pain, with lower pain perception (higher scores) in young and middle-aged individuals compared to the older ones (p = 0.004 and p = 0.010, respectively) (Tables 1, 2 and 3).

Regarding gender, men showed better recovery in general health status (2nd and 4th month, p = 0.029) and social aspects (2nd and 6th month, p = 0.040). Women showed improvements in pain (2nd and 4th month, p = 0.038), vitality (4th and 6th month, p = 0.035), and mental health (2nd and 6th month, p = 0.005). When comparing the groups, the men showed higher averages in virtually all domains. Significant differences remained throughout the three months for functional capacity, vitality, and mental health (p < 0.001 in all). Regarding limitations related to physical aspects, pain, general health status, social aspects, and emotional aspects, male superiority was statistically significant in most areas assessed, with p-values<0.05 (Tables 1, 2 and 3).

Race/color modulated recovery time: whites improved social aspects and mental health earlier (2nd and 4th month, p = 0.025 and p = 0.035, respectively) and later (4th and 6th month for social aspects, p = 0.011). Likewise, brown children concentrated their evolution between the 2nd and 6th month in general health status (p = 0.022) and mental health (p = 0.002). In the 6th month, brown individuals outperformed white individuals in the domain vitality (p = 0.014) (Tables 2 and 3).

In the second layer, the frequency of physical activity influenced the pattern of improvement. Sedentary or slightly active individuals (0 to 3 days/week) showed improvements between the 2nd and 6th month in limitations related to physical aspects (p = 0.019), vitality (p = 0.010), social aspects (p = 0.022), and mental health (p = 0.002). Those who were more active (4 to 7 days) showed a punctual gain in social aspects (2nd and 4th month, p = 0.018), but maintained averages consistently higher than those who were less active in functional capacity in the 2nd (p = 0.010), 4th (p = 0.010), and 6th month (p = 0.042); limitations due to physical aspects in the 2nd month (p = 0.009); and pain in the 2nd (p = 0.028) and 4th month (p = 0.021) (Tables 1, 2 and 3).

Diet and sun exposure also impacted the trajectory. Low intake of fruits and vegetables (0 to 3 days/week) showed improvement in mental health, especially between the 2nd and 6th month (p = 0.008). Among those who consumed more frequently (4 to 7 days), there was an improvement in social aspects with a significant difference between the 2nd and 4th month (p = 0.046). Lower consumption of fried and processed foods was associated with improvements between the 2nd and 6th month in functional capacity (p = 0.026), vitality (p = 0.005), and mental health (p < 0.001). The social aspects domain showed a difference between the 2nd and 4th month (p = 0.036). Those who consumed fried and processed foods 0 to 3 days per week showed better scores in the social aspects domain at month 6 (p = 0.025) (Tables 1, 2 and 3).

Participants with less sun exposure (0 to 3 days/week) showed improvements observed between the 2nd and 6th month for vitality (p = 0.005), social aspects (p = 0.010) and mental health (p = 0.001), and between the 2nd and 4th month for general health status (p = 0.030) and social aspects (p = 0.046). Those who were exposed to the sun 0 to 3 days a week also showed higher averages in social aspects in the 6th month (p = 0.010) (Tables 2 and 3).

Sleep difficulties showed differences between the 2nd and 4th month in the domains of pain (p = 0.036), social aspects (p = 0.009), and mental health (p < 0.001), in addition to the difference between the 2nd and 6th month for general health status (p = 0.030). Among non-smokers, differences were observed in the following domains: pain (2nd and 4th month, p = 0.029); vitality (2nd and 6th month, p = 0.022); social aspects (2nd and 4th month, p = 0.014 and 4th and 6th month, p = 0.008); and mental health (2nd and 6th month, p = 0.001) (Tables 2 and 3).

Individuals who did not consume alcohol showed positive progress in pain management, with significant differences between the 2nd and 4th month in the pain (p = 0.012) and social aspects (p = 0.012) domains. Between the 2nd and 6th month, the differences were in the domains of vitality (p = 0.010), social aspects (p = 0.031), and mental health (p = 0.006) (Tables 2 and 3).

In the third layer, social support was relevant. Living alone or with one other person showed significant improvement only in mental health (2nd and 6th month, p = 0.007). Those who lived with two or more people showed improvements in vitality (2nd and 6th month, p = 0.016) and mental health (2nd and 6th month, p = 0.005). In the social aspects domain, the difference occurred between the 2nd and 4th month (p = 0.005) (Tables 2 and 3).

Living with people from the at-risk group was associated with improvements in vitality between the 4th and 6th month (p = 0.021) and in social aspects between the 2nd and 4th month (p = 0.031). Among those who did not live with people in the at-risk group, statistically significant differences were observed between the 2nd and 4th month for the vitality (p = 0.013) and mental health (p = 0.033) domains, as well as between the 2nd and 4th month (p = 0.039) and between the 4th and 6th month (p = 0.036) for the general health status domain (Tables 2 and 3).

In the fourth layer, living conditions, participants who lived in places with public water supply showed evident differences in the vitality domain between the 2nd and 6th month (p = 0.006), social aspects between the 2nd and 4th month (p = 0.010) and mental health between the 2nd and 4th month (p = 0.022). Those who had access to public water supply showed better averages in the emotional aspects domain in the 6th month (p = 0.020) (Tables 2 and 3).

Regarding the type of sewage disposal, individuals residing in areas with a sewage system showed significant improvements in vitality between the 2nd and 6th month (p = 0.012), social aspects between the 2nd and 4th month (p = 0.045), and mental health between the 4th and 6th month (p = 0.033) (Tables 2 and 3).

In the fifth layer, participants with a monthly family income of less than three minimum wages showed improvement with significant differences in social aspects (the 2nd and 4th month, p = 0.022) and mental health (2nd and 6th month, p = 0.022). For participants with income above three minimum wages, differences were observed in vitality (4th and 6th month, p = 0.041) and mental health (2nd and 6th month, p = 0.012) (Tables 2 and 3).

## DISCUSSION

The results show that the SDHs influence the QoL of patients diagnosed with COVID-19 over time, confirming that the trajectory of improvement depends not only on clinical resolution, but also on structural and behavioral conditions. The predominance of young adults aligns with the change of the epidemiological profile of COVID-19^(15)^, in contrast to the higher average age observed at the beginning of the pandemic^(16)^. This may have contributed to the observed functional recovery, in contrast to the poorer quality of life reported in older individuals after infection^(17)^.

Regarding sex, the higher proportion of women reflects female engagement in healthcare practices and COVID-19 testing^(18)^. However, men showed better recovery in physical and social aspects, while women obtained better results in mental health. This disparity suggests differences in coping and the psychosocial impact of the pandemic between sexes^(19)^.

In terms of race/color, the predominance of people who self-identify as mixed-race reflects the Brazilian demographic composition^(20)^ and findings from previous studies with populations affected by COVID-19^(20,21,22)^. The distinction in recovery between whites (better in mental health) and mixed-race (brown) individuals (better in physical aspects) reinforces the weight of structural inequalities and social stress faced by racialized groups in Brazil^(22)^.

Lifestyle has emerged as a central factor. High levels of sedentary behavior reflect the impacts of social isolation during the pandemic^(23)^. However, regular physical activity has proven to be protective for vitality and mental health^(24)^. Similar patterns were observed in diet and sleep: consumption of healthy foods was associated with better psychosocial outcomes^(25)^ while sleep disturbances and low sun exposure were associated with worse indicators in several domains, highlighting the relevance of sunlight for vitamin D synthesis and emotional balance, and the impact of confinement on circadian rhythm and well-being^(26)^. These findings reinforce the role of lifestyle-related behaviors, the intermediate layer of the Dahlgren and Whitehead model, in modulating quality of life during the post-COVID-19 period.

Smoking showed low prevalence, corroborating studies that point to a greater concern with respiratory diseases during the health crisis^(27)^. The absence of smoking and non-consumption of alcohol acted as protective factors for overall quality of life, contradicting trends of increased consumption during the pandemic^(23,28)^.

Within social and community networks, cohabitation with a greater number of people or at-risk groups negatively impacted vitality and pain, reflecting the difficulty of isolation and the overload of care in those environments^(20,29)^. In terms of housing conditions, access to basic sanitation (water and sewage) proved fundamental for recovery in almost all areas of quality of life^(30)^. Low family income is a significant indicator of vulnerability that can increase the risk of exposure and hinder access to essential resources^(31)^. Nevertheless, this study revealed that families earning up to three minimum wages showed better results in physical and social aspects, while those with higher incomes stood out only in the vitality domain. This nuance suggests that income, while a critical SDH, may have complex interactions with other factors of protection or vulnerability post-COVID-19.

Interpreting the findings in light of the Dahlgren and Whitehead model^(6)^ allows us to understand that the SDH act interdependently in the recovery of QoL after COVID-19^(32)^. Individual factors, such as age, sex, and race/color, interact with lifestyle-related behaviors, social and community networks, as well as housing and income conditions, distinctly influencing the physical and mental domains of QoL throughout the follow-up period. In this regard, it was observed that lifestyle, housing conditions, income, and support networks modulated the recovery trajectory, showing that individuals exposed to more favorable social contexts presented better evolution in certain domains of the SF-36. These findings reinforce the idea that post-COVID-19 recovery is not homogeneous, being permeated by social inequalities that influence the rehabilitation process.

The positive evolution of SF-36 scores over the six months is consistent with international studies^(14,33)^, indicating a progressive restoration of health. Monitoring over a six-month period was essential to determine the impacts of the SDH over time, as well as to observe the trend in the QoL evolution of individuals affected by COVID-19. The influence of the SDHs reinforces the need for intersectoral strategies focusing primarily on the SDH that have a direct impact on individuals’ QoL. In Primary Care, Nursing should lead the planning of care that integrates the promotion of healthy habits and the strengthening of support networks, considering regional inequalities to ensure equitable rehabilitation^(12)^.

It is important to acknowledge the limitations of this study. The geographical restriction to the states of Ceará and Maranhão limits the generalization of the findings at the national level. Furthermore, potential intervening factors during follow-up, such as COVID-19 reinfection, worsening of clinical status, access to rehabilitation services, or changes in participants’ living conditions, which could influence the observed quality of life scores, were not controlled for. The discontinuation of participants throughout the follow-up period (from 391 to 179 participants), although expected in the sample size calculation, may have weakened the strength of some associations. Despite this, the findings provide relevant insights for public management and clinical practice in the post-pandemic context.

## CONCLUSION

It is concluded that the QoL of individuals in post-COVID-19 condition showed progressive and statistically significant improvement over time, with this trajectory being intrinsically influenced by the SDHs. Factors related to lifestyle, housing conditions, income, and support networks modulated the recovery of physical and mental domains, demonstrating that the rehabilitation process does not occur homogeneously, but is permeated by social inequalities that impact the evolution of QoL.

These findings reinforce the need for nursing and public management to implement longitudinal strategies that go beyond the clinical management of the post-COVID-19 condition. In this regard, intersectoral actions aimed at promoting healthy habits, encouraging physical activity, providing health education on nutrition and sleep, strengthening social support networks, as well as public policies aimed at improving housing conditions, basic sanitation, and equitable access to health and rehabilitation services stand out.

As a scientific contribution, this study advances the longitudinal analysis of the influence of SDH on post-COVID-19 QoL, highlighting how different layers of the Dahlgren and Whitehead model interact in the recovery process. These results contribute to a broader understanding of inequalities in the post-pandemic context and provide support for the planning of Nursing interventions in Primary Health Care, guided by equity and health promotion. It is recommended that the study be replicated in other contexts to broaden the understanding of the pandemic’s impact on the Brazilian population and to support health policies guided by equity.

## Data Availability

The entire dataset supporting the results of this study was published in the article itself.
