# Supplementary material for: Post-COVID-19 social determinants of health and quality of life: a longitudinal study
Source: Rev Esc Enferm USP. 2026 Jun 12;60:e20250565. doi: 10.1590/1980-220X-REEUSP-2025-0565en (PMC13268353; doi:10.1590/1980-220X-REEUSP-2025-0565en)
Supplement: Translation

# Translation: Determinantes sociais da saúde e qualidade de vida pós-COVID-19: estudo longitudinal

**DOI:** 10.1590/1980-220X-REEUSP-2025-0565pt

**Authors:** Sabrina de Souza Gurgel Florencio, Francisca Elisângela Teixeira Lima, Glaubervania Alves Lima, Maria Gabriela Miranda Fontenele, Lívia Maia Pascoal, Lilia Jannet Saldarriaga Sandoval, Paulo César de Almeida, Gilvan Ferreira Felipe, Maria Williany Silva Ventura, Brena Shellem Bessa de Oliveira, Lorena Pinheiro Barbosa

**Affiliations:** 1 Universidade Federal do Ceará, Fortaleza, CE, Brasil.; 2 Universidade Federal do Maranhão, Imperatriz, MA, Brasil.; 3 Universidad Nacional de Tumbes, Tumbes, Peru.; 4 Universidade Estadual do Ceará, Fortaleza, CE, Brasil.; 5 Universidade da Integração Internacional da Lusofonia Afro-Brasileira, Redenção, CE, Brasil.

**Keywords:** COVID-19, Determinantes Sociais da Saúde, Qualidade de Vida, Enfermagem, Saúde Pública

## Abstract

**Objetivo:**

Verificar a associação entre os determinantes sociais da saúde e os domínios de qualidade de vida em pessoas em condição pós-COVID-19.

**Métodos:**

Estudo longitudinal, realizado com 179 pessoas dos estados do Ceará e Maranhão, acompanhadas por seis meses após diagnóstico confirmado de COVID-19. A coleta de dados ocorreu por meio do *link* do *software* de monitoramento de pacientes com COVID-19, enviado mensalmente via aplicativo de mensagens telefônicas. Consideram-se as variáveis relativas aos determinantes sociais da saúde e aos domínios do questionário SF-36 para avaliação da qualidade de vida. Dados analisados mediante estatística descritiva e inferencial.

**Resultados:**

Mulheres apresentaram melhorias no domínio dor entre o 2º e 4º mês (p = 0,038), vitalidade entre o 4º e 6º (p = 0,035) e saúde mental entre o 2º e 6º mês (p = 0,005). Homens apresentaram melhor recuperação no estado geral de saúde no 2º e 4º mês (p = 0,029) e nos aspectos sociais no 2º e 6º mês (p = 0,040).

**Conclusão:**

Observou-se melhora progressiva da qualidade de vida ao longo do seguimento, com diferenças entre homens e mulheres nos domínios dor, vitalidade, saúde mental, estado geral de saúde e aspectos sociais.

## INTRODUÇÃO

A pandemia da COVID-19 desencadeou uma crise sanitária global cujos impactos ultrapassaram a fase aguda da infecção, gerando repercussões prolongadas nas condições físicas, emocionais e sociais das pessoas acometidas, configurando a condição pós-COVID-19^(1,2)^. Esse cenário afetou diretamente a qualidade de vida (QV) da população, entendida como a percepção do indivíduo sobre sua posição na vida, no contexto cultural e dos valores em que está inserido^(3)^. Na pandemia, a QV tornou-se vulnerável às alterações nos hábitos cotidianos, condições de trabalho, moradia, alimentação e acesso aos serviços de saúde^(4)^.

Tais elementos integram os Determinantes Sociais da Saúde (DSS), fatores sociais, econômicos, culturais, étnico-raciais, psicológicos e comportamentais que influenciam o risco de adoecimento e mortalidade nas populações^(5)^. Para compreender essa interação, este estudo adota o modelo de Dahlgren e Whitehead^(6)^, que organiza os DSS em camadas concêntricas, desde fatores individuais, estilos de vida, redes sociais e comunitárias, até condições de vida e trabalho e, finalmente, os macrodeterminantes socioeconômicos. Essa estrutura evidencia a complexa interação entre o indivíduo e seu contexto social e ambiental na determinação da saúde.

Ao atuar sobre cada uma dessas camadas, a pandemia de COVID-19 funcionou como um potente intensificador das desigualdades estruturais já descritas no modelo de Dahlgren e Whitehead, tornando ainda mais evidentes as vulnerabilidades preexistentes. Nesse contexto, grupos com menor nível socioeconômico, saneamento inadequado, moradia precária, acesso limitado à alimentação saudável e aos serviços de saúde foram desproporcionalmente afetados^(7)^. Tais desigualdades aumentaram o risco de infecção, mortalidade e comprometeram a QV dos sobreviventes. Embora a literatura tenha investigado a QV pós-COVID-19^(8,9)^, ainda são limitados os estudos que analisam a influência dos DSS na evolução da QV ao longo do tempo, especialmente em investigações longitudinais realizadas em estados do Nordeste brasileiro como o Ceará e o Maranhão, marcados por desigualdades socioeconômicas historicamente mais pronunciadas.

No Brasil, a pandemia evidenciou lacunas históricas no acesso a direitos sociais básicos. A implementação de medidas emergenciais, como o Programa de Auxílio Emergencial (PAE), benefício financeiro temporário criado pelo governo federal para reduzir os impactos econômicos da pandemia entre populações socialmente vulneráveis, embora necessária, também expôs limitações estruturais na capacidade de resposta e proteção social do Estado^(10)^. Nesse cenário, a avaliação dos DSS e sua influência na QV pós-COVID-19 é fundamental para identificar grupos mais impactados e subsidiar políticas públicas mais equitativas e eficazes.

Nesse contexto, torna-se necessário avaliar como os determinantes sociais da saúde influenciam a qualidade de vida das pessoas após a infecção. A avaliação da QV, por meio de instrumentos sensíveis a mudanças temporais como o *Short-Form Health Survey* (SF-36), torna-se fundamental para identificar vulnerabilidades persistentes^(11)^. O delineamento longitudinal constitui um diferencial metodológico relevante, pois permite acompanhar a trajetória de recuperação ao longo do tempo, capturando variações nos domínios de QV e a influência dos DSS em diferentes momentos do período pós-COVID-19, algo que estudos transversais não conseguem evidenciar. Assim, é preciso compreender quais DSS influenciam a QV de pessoas em condição pós-COVID-19. Essa compreensão contribui para a produção de conhecimento científico e para o planejamento de ações em saúde que visem à prevenção de agravos, reabilitação funcional e promoção da saúde em uma perspectiva integral e equitativa^(12)^.

Portanto, o presente estudo tem como objetivo verificar a associação entre os DSS e os domínios de QV em pessoas em condição pós-COVID-19. Os resultados podem subsidiar enfermeiros e gestores de saúde na elaboração de estratégias de cuidado longitudinal que considerem as iniquidades regionais, orientando intervenções voltadas à reabilitação funcional, promoção da saúde mental e fortalecimento do suporte social no contexto pós-pandêmico.

## MÉTODO

### Tipo de Estudo

Trata-se de um estudo longitudinal, analítico e quantitativo, realizado nos estados do Ceará e Maranhão, regiões que apresentaram elevada incidência de COVID-19 durante a pandemia. O acompanhamento ocorreu durante seis meses, com envio mensal de formulários de monitoramento e aplicação do SF-36 2º, 4º e 6º mês, para indivíduos diagnosticados com COVID-19.

### Protocolo do Estudo

A população de referência foi composta por 573.555 adultos e idosos (≥ 20 anos), com sintomas gripais notificados no Sistema de Informação de Vigilância da Gripe (SIVEP-GRIPE) e/ou no e-SUS Notifica, sendo 367.265 do Ceará e 206.290 do Maranhão. Considerando um erro amostral de 7% e intervalo de confiança de 95%, estimou-se uma amostra mínima de 179 participantes.

Foram incluídos na amostra indivíduos com diagnóstico confirmado de COVID-19 e número de telefone celular com acesso à internet registrado nos referidos sistemas de informação das Secretarias de Saúde de cada estado. Foram excluídos aqueles com limitação cognitiva autorreferida ou que não responderam após três tentativas de contato. Das 391 pessoas elegíveis que responderam ao primeiro formulário, houve 212 desistências, motivadas principalmente por falta de resposta após novas tentativas de contato (n = 168), recusa em continuar participando (n = 28) e mudança ou inativação do número de telefone (n = 16), resultando na amostra final de 179 participantes, sendo 95 do Ceará e 84 do Maranhão.

Para coleta de dados, utilizou-se o *Software* de monitoramento de pacientes com COVID-19, desenvolvido e validado no Brasil^(13)^, o qual contém dois instrumentos: um formulário de caracterização sociodemográfica e estilo de vida, contemplando os DSS (incluindo variáveis de idade, sexo, raça/cor, atividade física, hábitos alimentares, exposição solar, qualidade do sono, tabagismo, etilismo, composição domiciliar, convivência com grupos de risco, saneamento básico e renda familiar, organizados conforme as camadas do modelo de Dahlgren e Whitehead); e o *Short-Form Health Survey* (SF-36), traduzido e validado para o Brasil, composto por 36 itens distribuídos em oito domínios (capacidade funcional, aspectos físicos, dor, estado geral de saúde, vitalidade, aspectos sociais, aspectos emocionais e saúde mental)^(11)^.

A coleta ocorreu entre junho de 2021 e junho de 2022. Inicialmente, foram obtidos dados dos sistemas SIVEP-GRIPE e e-SUS Notifica, que registram casos suspeitos e confirmados de COVID-19 no país. As fichas de notificação foram preenchidas por profissionais da saúde e consolidadas pelas Vigilâncias Epidemiológicas das Secretarias Estaduais da Saúde (SESA) do Ceará e Maranhão.

Após autorização das SESA, os dados foram exportados para o software de monitoramento, que gerava um link individual para cada participante. Esse link, acompanhado de convite para participação, Termo de Consentimento Livre e Esclarecido (TCLE) e formulário mensal de acompanhamento, foi enviado por WhatsApp, aplicativo de mensagens amplamente utilizado no Brasil, o que pode ter favorecido a inclusão de participantes com maior acesso à internet e letramento digital, constituindo uma limitação do método de recrutamento. O SF-36 foi aplicado no segundo, quarto e sexto mês de seguimento. Esses intervalos foram definidos com base na literatura sobre condição pós-COVID-19, que aponta os primeiros seis meses como o período de maior variabilidade na recuperação clínica e funcional, com avaliações bimestrais para capturar mudanças progressivas nos domínios de QV^(2,14)^. O prazo para resposta foi de três dias.

### Análise dos Dados

Os dados foram analisados no programa *Statistical Package for the Social Sciences* (SPSS), versão 20.0, licença 10101131007. As variáveis foram descritas por meio de frequências absolutas, relativas, médias e desvios padrão. A normalidade dos dados foi avaliada pelo teste de *Kolmogorov-Smirnov*.

Diante da distribuição não normal, aplicou-se o *teste de Friedman*, seguido do *teste de Conover* para comparações múltiplas entre o 2º, 4º e 6º mês, visando verificar a evolução dos domínios do SF-36 e sua associação com os determinantes sociais da saúde. Para comparação entre grupos independentes, utilizaram-se os testes *Kruskal-Wallis* e *Mann-Whitney*. Nas variáveis com diferenças significativas pelo Kruskal-Wallis, realizou-se teste post hoc de Dunn com correção de Bonferroni. O nível de significância adotado foi p < 0,05.

### Aspectos Éticos

O estudo foi aprovado pelo Comitê de Ética em Pesquisa, sob parecer nº 4.278.495/2020 e identificador CAAE 36193820.1.0000.5054.

## RESULTADOS

Participaram do estudo 179 indivíduos, predominantemente mulheres (58,7%), com média de idade de 37,4 ± 12,6 anos e autodeclarados pardos (56,4%). Observou-se baixa adesão à atividade física regular (81,0%), consumo insuficiente de frutas e verduras (52,5%), alta ingestão de alimentos fritos e industrializados (73,2%), baixa exposição solar (60,9%) e dificuldade para dormir (55,3%). O consumo de álcool foi relatado por 44,1% e 4,5% eram tabagistas. A maioria residia com duas ou mais pessoas (95,0%), conviviam com indivíduos do grupo de risco (50,3%), possuía acesso à rede geral de distribuição de água (93,9%), rede de esgoto (67,0%) e viviam com renda mensal de até três salários mínimos (59,8%) (Tabela 1).

**Tabela 1:** Associação dos determinantes sociais da saúde de pessoas com COVID-19 com as médias dos domínios capacidade funcional e limitações por aspectos físicos do instrumento SF-36, comparando o 2º, 4º e 6º mês – Fortaleza, CE, Brasil, 2023.

Domínios SF-36	Total			Capacidade funcional média (±DP)					Limitações por aspectos físicos média (±DP)			
	n	%		2º mês	4º mês	6º mês	p*		2º mês	4º mês	6º mês	p*
**1ª camada – Idade, sexo e fatores hereditários**												
**Idade**												
20–39	114	63,7		83,4 (±20,4)	83,7 (±19,2)	84,2 (±18,4)	0,905		76,5 (±35,7)	82,2 (±30,9)	84,2 (±27,6)	0,070
40–59	56	31,3		75,8 (±22,8)	76,9 (±23,6)	78,8 (±21,2)	**0,038**		71,4 (±37,9)	73,2 (±38,6)	74,5 (±36,1)	0,645
60–75	9	5,0		66,6 (±25,1)	68,3 (±23,3)	69,4 (±16,2)	0,747		66,6 (±45,0)	75,0 (±39,5)	69,4 (±46,3)	0,819
**p-valor** ^§^				**0,008**	**0,034**	**0,027**			0,634	0,432	0,254	
Sexo												
Masculino	105	58,7		87,4 (±17,0)	86,6 (±18,5)	88,1 (±17,1)	0,591		80,0 (±34,8)	88,5 (±25,5)	89,8 (±23,0)	**0,038**
Feminino	74	41,3		75,1 (±23,4)	76,6 (±22,1)	77,4 (±19,9)	0,331		70,4 (±37,7)	72,3 (±37,6)	73,8 (±35,4)	0,467
**p-valor** ^†^				**<0,001**	**0,001**	**<0,001**			**0,042**	**0,002**	**0,001**	
Raça/Cor												
Branca	57	31,8		79,3 (±26,4)	80,5 (±22,2)	83,0 (±20,2)	0,560		74,5 (±39,1)	76,3 (±33,8)	77,6 (±32,2)	0,672
Preta	21	11,7		80,0 (±21,8)	78,8 (±23,6)	76,1 (±20,9)	0,115		71,4 (±35,6)	70,2 (±40,7)	80,9 (±34,3)	0,353
Parda	101	56,4		80,7 (±18,9)	81,4 (±20,3)	82,3 (±18,7)	0,383		75,0 (±36,0)	82,4 (±32,5)	81,9 (±31,2)	**0,024**
**p-valor** ^§^				0,745	0,951	0,307			0,754	0,208	0,549	
**2ª camada – Estilo de vida dos indivíduo**s												
**Atividade física/semana**												
0 a 3 dias	145	81,0		78,6 (±21,9)	79,2 (±21,6)	80,5 (±19,7)	0,236		71,5 (±37,8)	79,3 (±34,1)	81,5 (±30,7)	**<0,001**
4 a 7 dias	34	19,0		86,9 (±20,1)	87,9 (±18,3)	87,3 (±17,7)	0,500		86,7 (±29,6)	77,9 (±34,1)	75,7 (±36,1)	**0,026**
**p-valor** ^†^				**0,010**	**0,010**	**0,042**			**0,009**	0,832	0,583	
**Consumo de frutas e de verduras/semana**												
0 a 3 dias	94	52,5		78,3 (±21,6)	79,8 (±20,8)	80,7 (±19,7)	0,179		76,5 (±34,5)	78,9 (±32,4)	81,1 (±29,9)	0,300
4 a 7 dias	85	47,5		82,2 (±21,9)	81,9 (±21,7)	83,0 (±19,2)	0,805		72,0 (±39,2)	79,1 (±35,9)	79,7 (±33,9)	0,121
**p-valor** ^†^				0,071	0,280	0,391			0,760	0,686	0,810	
**Consumo de frituras e industrializados/semana**												
0 a 3 dias	131	73,2		79,3 (±22,5)	81,1 (±21,9)	83,0 (±19,2)	**0,026**		75,0 (±35,7)	79,0 (±33,9)	79,9 (±31,5)	0,082
4 a 7 dias	48	26,8		82,5 (±19,5)	80,1 (±19,3)	78,6 (±20,1)	0,136		72,9 (±39,8)	79,1 (±34,7)	81,7 (±32,9)	0,353
**p-valor** ^†^				0,420	0,912	0,119			0,818	0,482	0,510	
**Exposição ao sol/semana**												
0 a 3 dias	109	60,9		79,0 (±22,0)	82,1 (±19,5)	82,1 (±18,9)	0,110		78,8 (±32,6)	81,6 (±31,1)	81,8 (±30,2)	0,202
4 a 7 dias	70	39,1		82,0 (±21,4)	78,8 (±23,6)	81,3 (±20,4)	0,580		67,5 (±41,7)	75,0 (±38,0)	78,2 (±34,2)	0,198
**p-valor** ^†^				0,254	0,636	0,922			0,147	0,334	0,589	
**Dificuldade para dormir**												
Sim	99	55,3		77,4 (±22,8)	80,5 (±21,4)	79,9 (±20,7)	0,170		82,1 (±31,3)	85,0 (±29,9)	85,9 (±26,3)	0,133
Não	80	44,7		83,6 (±20,0)	81,3 (±21,0)	84,1 (±17,7)	0,591		68,1 (±39,7)	74,2 (±36,5)	76,0 (±35,1)	0,340
**p-valor** ^†^				**0,035**	0,748	0,244			**0,019**	**0,022**	0,059	
**Tabagismo**												
Sim	8	4,5		86,2 (±24,0)	90,6 (±10,5)	87,5 (±18,5)	0,953		100 (±00,0)	90,6 (±26,5)	100 (±00,0)	0,368
Não	171	95,5		79,9 (±21,7)	80,4 (±21,5)	81,5 (±19,5)	0,427		73,2 (±37,2)	78,5 (±34,3)	79,5 (±32,2)	**0,041**
**p-valor** ^†^				0,167	0,319	0,337			**0,019**	0,224	**0,043**	
**Etilismo**												
Sim	79	44,1		83,1 (±20,3)	83,2 (±19,9)	84,3 (±18,0)	0,756		76,8 (±35,0)	82,2 (±30,7)	85,1 (±27,8)	0,164
Não	100	55,9		77,9 (±22,7)	79,0 (±22,1)	79,9 (±20,4)	0,160		72,5 (±38,1)	76,5 (±36,3)	76,7 (±34,3)	0,256
**p-valor** ^†^				0,136	0,274	0,211			0,461	0,388	0,126	
**3ª camada – Redes sociais e comunitárias**												
**Quantidade de pessoas que reside**												
Mora só ou uma	9	5,0		75,5 (±23,7)	76,1 (±25,0)	78,8 (±26,1)	0,857		72,2 (±42,2)	72,2 (±44,0)	91,6 (±17,6)	0,779
≥ 2	170	95,0		80,4 (±21,7)	81,1 (±21,0)	82,0 (±19,1)	0,481		74,5 (±36,6)	79,4 (±33,5)	79,8 (±32,3)	**0,043**
**p-valor** ^†^				0,471	0,551	0,970			0,819	0,802	0,351	
**Reside com pessoas do grupo de risco**												
Sim	90	50,3		79,4 (±21,6)	80,7 (±20,7)	82,3 (±20,0)	0,819		73,3 (±37,8)	80,2 (±33,6)	80,8 (±32,4)	**0,018**
Não	89	49,7		80,9 (±22,0)	80,9 (±21,8)	81,3 (±19,0)	0,114		75,5 (±35,9)	77,8 (±34,6)	80,0 (±31,3)	0,416
**p-valor** ^†^				0,525	0,678	0,717			0,687	0,597	0,815	
**4ª camada – Condições de vida**												
**Abastecimento de água**												
Rede de distribuição	168	93,9		81,1 (±20,2)	81,5 (±21,0)	82,3 (±19,1)	0,387		74,1 (±36,9)	80,0 (±33,5)	81,5 (±30,4)	**0,017**
Outro	11	6,1		66,8 (±37,7)	70,4 (±22,6)	74,0 (±24,5)	0,882		79,5 (±35,0)	63,6 (±39,3)	63,6 (±46,5)	0,444
**p-valor** ^†^				0,508	0,054	0,276			0,642	0,119	0,212	
**Escoadouro de esgoto**												
Rede de esgoto	120	67,0		79,7 (±20,7)	81,5 (±19,6)	81,5 (±19,3)	0,526		73,9 (±36,8)	80,6 (±33,2)	81,6 (±29,6)	**0,042**
Outro	59	33,0		81,2 (±24,0)	79,5 (±24,2)	82,5 (±19,9)	0,853		75,4 (±36,9)	75,8 (±35,6)	77,9 (±35,9)	0,764
**p-valor** ^†^				0,293	0,861	0,465			0,537	0,446	0,922	
**5ª camada – Condições socioeconômicas, culturais e ambientais gerais**												
**Renda familiar**												
< 3 salários mínimos	107	59,8		78,8 (±22,3)	79,5 (±21,4)	79,7 (±20,4)	0,598		73,5 (±37,7)	80,3 (±32,5)	81,3 (±31,6)	**0,027**
> 3 salários mínimos	72	40,2		82,2 (±20,9)	82,8 (±20,9)	85,0 (±17,6)	0,532		75,6 (±35,5)	77,0 (±36,2)	79,1 (±32,2)	0,856
**p-valor** ^†^				0,311	0,299	0,142			0,843	0,711	0,510	

Legenda: *Teste de Frideman/

^§^Kruskal-Wallis/

^†^Mann-Whitney.

A análise longitudinal da associação entre os DSS e os domínios do SF-36 revelou variações significativas ao longo do 2º, 4º e 6º meses de acompanhamento. Essas diferenças ocorreram conforme características sociodemográficas, estilos de vida, condições ambientais e redes sociais (Tabelas 1, 2 e 3).

**Tabela 2:** Associação dos determinantes sociais da saúde de pessoas com COVID-19 com as médias dos domínios dor, estado geral de saúde e vitalidade do instrumento SF-36, comparando o 2º, 4º e 6º mês – Fortaleza, CE, Brasil, 2023.

Domínios SF-36	Dor média (±DP)					Estado geral de saúde média (±DP)					Vitalidade média (±DP)			
	2º mês	4º mês	6º mês	p*		2º mês	4º mês	6º mês	p*		2º mês	4º mês	6º mês	p*
1ª camada – Idade, sexo e fatores hereditários														
Idade														
20–39	72,5 (±23,1)	73,5 (±22,4)	74,4 (±22,4)	0,514		64,4 (±20,5)	65,5 (±20,6)	66,4 (±21,2)	0,507		53,7 (±18,9)	54,3 (±22,0)	56,4 (±19,9)	0,246
40–59	66,4 (±24,4)	70,5 (±23,2)	72,0 (±23,6)	**0,015**		64,0 (±17,3)	68,7 (±18,3)	66,1 (±19,2)	0,096		56,8 (±19,4)	60,0 (±19,4)	62,2 (±19,0)	**0,006**
60–75	60,3 (±18,3)	65,1 (±27,5)	53,0 (±11,0)	0,485		52,3 (±8,9)	54,7 (±10,9)	55,3 (±12,2)	0,678		52,2 (±12,0)	53,8 (±13,8)	56,6 (±13,2)	0,482
**p-valor** ^§^	0,155	0,555	**0,016**			0,090	0,074	0,152			0,563	0,278	0,217	
**Sexo**														
Masculino	76,3 (±22,5)	75,8 (±22,4)	78,0 (±21,4)	0,555		68,2 (±17,6)	72,0 (±18,9)	71,1 (±18,3)	**0,045**		61,4 (±19,3)	64,3 (±20,2)	63,9 (±18,6)	0,161
Feminino	65,6 (±23,2)	69,5 (±23,0)	68,8 (±23,1)	**0,035**		60,5 (±19,8)	61,7 (±19,2)	62,0 (±20,9)	0,839		49,8 (±16,9)	50,3 (±19,6)	54,3 (±19,1)	**0,009**
**p-valor** ^†^	**0,003**	0,068	**0,009**			**0,013**	**<0,001**	**0,004**			**<0,001**	**<0,001**	**0,001**	
**Raça/Cor**														
Branca	64,1 (±26,2)	69,6 (±24,4)	70,9 (±24,5)	0,036		62,2 (±22,8)	64,2 (±23,2)	63,2 (±22,4)	0,064		53,7 (±21,1)	54,0 (±22,4)	53,5 (±20,5)	0,856
Preta	72,5 (±22,4)	65,5 (±24,0)	75,3 (±21,5)	0,220		62,2 (±16,3)	63,8 (±19,2)	62,2 (±12,7)	0,334		55,0 (±18,5)	52,1 (±23,6)	53,8 (±18,9)	0,776
Parda	72,8 (±21,6)	74,9 (±21,6)	73,0 (±22,1)	0,073		64,8 (±17,6)	67,4 (±17,6)	67,9 (±20,2)	0,035		55,0 (±17,6)	58,1 (±19,5)	61,8 (±18,3)	**<0,001**
**p-valor** ^§^	0,131	0,199	0,825			0,771	0,603	0,161			0,925	0,402	0,024	
**2ª camada – Estilo de vida dos indivíduos**														
**Atividade física/semana**														
0 a 3 dias	68,2 (±23,3)	70,2 (±22,7)	71,7 (±23,0)	**0,048**		62,8 (±18,9)	64,6 (±19,3)	64,7 (±19,8)	0,215		53,3 (±19,0)	54,8 (±21,5)	57,6 (±19,7)	**0,015**
4 a 7 dias	77,7 (±22,7)	80,1 (±22,3)	76,3 (±21,8)	0,530		67,4 (±20,7)	72,1 (±20,4)	70,1 (±21,9)	0,512		60,1 (±17,1)	61,4 (±18,0)	60,8 (±18,4)	0,539
**p-valor** ^†^	**0,028**	**0,021**	0,312			0,257	0,059	0,159			0,050	0,125	0,402	
**Consumo de frutas e de verduras/semana**														
0 a 3 dias	69,9 (±23,0)	71,6 (±21,9)	72,1 (±22,8)	0,174		61,1 (±20,0)	64,4 (±19,8)	62,9 (±20,7)	0,215		54,2 (±18,2)	55,7 (±21,8)	58,2 (±19,7)	0,056
4 a 7 dias	70,2 (±24,1)	72,6 (±24,0)	73,1 (±22,9)	0,208		66,5 (±18,1)	67,8 (±19,5)	68,8 (±19,5)	0,589		55,1 (±19,4)	56,4 (±20,1)	58,2 (±19,3)	0,150
**p-valor** ^†^	0,831	0,610	0,699			0,084	0,278	0,069			0,754	0,871	0,853	
**Consumo de frituras e industrializados/semana**														
0 a 3 dias	70,4 (±23,4)	72,9 (±23,0)	73,4 (±22,2)	0,168		64,4 (±19,1)	67,2 (±18,5)	67,3 (±19,7)	0,235		55,1 (±18,4)	56,6 (±21,0)	59,7 (±19,0)	**0,006**
4 a 7 dias	69,1 (±23,7)	69,8 (±22,6)	70,5 (±24,3)	0,184		61,7 (±19,7)	62,7 (±22,4)	61,3 (±21,4)	0,344		53,2 (±19,8)	54,5 (±21,0)	54,2 (±20,2)	0,779
**p-valor** ^†^	0,754	0,278	0,557			0,548	0,539	0,097			0,500	0,397	0,139	
**Exposição ao sol/semana**														
0 a 3 dias	71,2 (±23,7)	73,1 (±22,6)	73,3 (±22,7)	0,168		63,1 (±19,1)	66,4 (±19,1)	65,8 (±20,2)	**0,041**		54,8 (±17,7)	57,4 (±21,0)	60,0 (±18,1)	**0,007**
4 a 7 dias	68,1 (±23,2)	70,6 (±23,4)	71,6 (±22,9)	0,214		64,6 (±19,5)	65,4 (±20,7)	65,6 (±20,5)	0,983		54,4 (±20,5)	54,0 (±20,9)	55,6 (±21,2)	0,593
**p-valor** ^†^	0,323	0,509	0,670			0,501	0,896	0,895			0,999	0,340	0,200	
**Dificuldade para dormir**														
Sim	66,8 (±24,5)	69,4 (±23,1)	70,1 (±23,0)	**0,011**		60,4 (±18,5)	63,6 (±20,1)	64,2 (±20,6)	**0,039**		51,3 (±19,6)	53,7 (±21,5)	54,7 (±20,4)	0,127
Não	74,0 (±21,7)	75,5 (±22,3)	75,6 (±22,2)	0,860		67,8 (±19,5)	68,9 (±18,9)	67,6 (±19,8)	0,279		58,8 (±16,9)	59,0 (±20,1)	62,6 (±17,3)	0,053
**p-valor** ^†^	**0,045**	0,099	0,118			**0,010**	0,074	0,368			**0,008**	0,060	**0,010**	
**Tabagismo**														
Sim	79,7 (±24,0)	67,5 (±24,9)	79,6 (±16,2)	0,228		69,8 (±20,1)	62,3 (±15,4)	63,3 (±26,0)	0,058		60,0 (±17,5)	61,8 (±17,5)	68,1 (±16,4)	**0,040**
Não	69,6 (±23,4)	72,3 (±22,9)	72,3 (±22,0)	**0,014**		63,4 (±19,2)	66,2 (±19,9)	65,8 (±20,1)	0,326		54,4 (±18,8)	55,8 (±21,1)	57,8 (±19,5)	**0,035**
**p-valor** ^†^	0,243	0,481	0,455			0,452	0,561	0,740			0,408	0,449	0,169	
**Etilismo**														
Sim	73,8 (±23,5)	72,6 (±22,7)	75,3 (±21,1)	0,618		66,2 (±19,5)	66,7 (±19,3)	69,1 (±18,2)	0,377		56,0 (±18,6)	55,6 (±21,5)	59,3 (±20,4)	0,374
Não	67,0 (±23,1)	71,7 (±23,2)	70,4 (±23,9)	0,008		61,7 (±18,9)	65,4 (±20,0)	63,1 (±21,5)	0,091		53,5 (±18,9)	58,5 (±20,6)	57,4 (±18,7)	**0,012**
**p-valor** ^†^	0,060	0,801	0,216			0,125	0,826	0,092			0,376	0,903	0,414	
**3ª camada – Redes sociais e comunitárias**														
**Quantidade de pessoas que reside**														
Mora só ou uma	63,0 (±22,6)	69,0 (±22,8)	72,5 (±24,9)	0,152		59,8 (±19,0)	63,4 (±20,0)	61,8 (±27,7)	0,401		53,3 (±16,0)	54,4 (±20,6)	65,0 (±22,0)	0,087
≥ 2	70,4 (±23,5)	72,3 (±22,8)	72,6 (±22,7)	0,091		63,9 (±19,3)	66,1 (±19,7)	65,9 (±19,9)	0,169		54,7 (±18,9)	56,2 (±21,1)	57,9 (±19,3)	**0,025**
**p-valor** ^†^	0,335	0,705	0,973			0,335	0,501	0,756			0,804	0,796	0,255	
**Reside com pessoas do grupo de risco**														
Sim	70,1 (±22,5)	72,2 (±22,1)	73,1 (±22,3)	0,273		64,6 (±20,1)	65,4 (±19,5)	67,3 (±19,8)	0,131		55,0 (±18,4)	53,7 (±21,2)	58,8 (±19,2)	**0,026**
Não	69,9 (±24,5)	72,0 (±23,8)	72,1 (±23,3)	0,115		62,7 (±18,4)	66,5 (±19,9)	64,1 (±20,8)	**0,026**		54,3 (±19,3)	58,4 (±20,7)	57,6 (±19,7)	**0,011**
**p-valor** ^†^	0,913	0,984	0,781			0,461	0,648	0,359			0,729	0,271	0,791	
**4ª camada – Condições de vida**														
**Abastecimento de água**														
Rede de distribuição	70,1 (±23,2)	71,8 (±22,9)	72,6 (±22,9)	0,062		63,6 (±19,3)	66,1 (±19,8)	65,7 (±20,4)	0,109		54,8 (±18,9)	56,6 (±21,0)	58,7 (±19,5)	**0,009**
Outro	68,1 (±27,7)	76,7 (±23,2)	77,2 (±20,9)	0,422		65,2 (±19,5)	64,0 (±18,2)	66,6 (±18,8)	1,000		51,3 (±17,3)	48,6 (±20,5)	50,9 (±17,8)	0,773
**p-valor** ^†^	0,887	0,450	0,966			0,675	0,711	0,873			0,613	0,233	0,204	
**Escoadouro de esgoto**														
Rede de esgoto	68,6 (±23,5)	71,6 (±23,0)	71,7 (±23,6)	**0,045**		63,6 (±19,2)	65,7 (±20,3)	65,6 (±20,1)	0,182		53,3 (±18,1)	53,7 (±19,8)	56,9 (±18,5)	**0,017**
Outro	72,9 (±23,2)	73,2 (±22,9)	74,5 (±21,0)	0,622		63,8 (±19,4)	66,7 (±18,5)	66,1 (±20,9)	0,640		57,3 (±20,0)	61,0 (±22,6)	61,1 (±21,1)	0,302
**p-valor** ^†^	0,216	0,623	0,434			0,881	0,746	0,866			0,155	**0,023**	0,195	
**5ª camada – Condições socioeconômicas, culturais e ambientais gerais**														
**Renda familiar**														
< 3 salários mínimos	70,8 (±23,6)	73,9 (±22,8)	73,4 (±23,4)	0,150		63,4 (±18,8)	65,1 (±19,5)	63,8 (±20,4)	0,468		54,8 (±19,6)	57,0 (±21,3)	57,7 (±20,1)	**0,048**
> 3 salários mínimos	68,9 (±23,4)	69,4 (±23,0)	71,4 (±22,0)	0,109		64,0 (±20,1)	67,3 (±19,9)	68,5 (±20,0)	0,130		54,4 (±17,5)	54,7 (±20,5)	59,0 (±18,5)	**0,034**
**p-valor** ^†^	0,593	0,197	0,455			0,664	0,433	0,123			0,972	0,473	0,656	

Legenda: *Teste de Frideman/

^§^Kruskal-Wallis/

^†^Mann-Whitney.

**Tabela 3:** Associação dos determinantes sociais da saúde de pessoas com COVID-19 com as médias dos domínios aspectos sociais, aspectos emocionais e saúde mental do instrumento SF-36, comparando o 2º, 4º e 6º mês – fortaleza, CE, brasil, 2023.

Domínios SF-36	Aspectos sociais média (±DP)					Aspectos emocionais média (±DP)					Saúde mental média (±DP)			
	2º mês	4º mês	6º mês	p*		2º mês	4º mês	6º mês	p*		2º mês	4º mês	6º mês	p*
**1ª camada – Idade, sexo e fatores hereditários**														
**Idade**														
20–39	70,5 (±24,7)	73,3 (±24,8)	72,5 (±24,4)	0,220		63,4 (±40,1)	71,6 (±37,1)	70,7 (±38,4)	**0,025**		60,3 (±18,6)	63,5 (±19,3)	65,5 (±20,3)	**0,013**
40–59	72,3 (±23,9)	77,0 (±25,4)	79,0 (±23,2)	**0,002**		76,7 (±37,0)	74,4 (±40,1)	75,5 (±38,9)	0,705		64,0 (±19,7)	65,2 (±21,5)	67,5 (±19,5)	0,204
60–75	63,8 (±20,1)	69,4 (±26,5)	79,1 (±22,5)	0,125		59,2 (±49,3)	62,9 (±48,4)	77,7 (±44,0)	0,368		53,3 (±12,6)	53,7 (±15,1)	60,4 (±15,9)	**0,042**
**p-valor** ^§^	0,508	0,479	0,212			0,056	0,596	0,537			0,163	0,240	0,482	
**Sexo**														
Masculino	77,5 (±22,5)	80,9 (±20,5)	81,9 (±21,4)	**0,014**		76,5 (±34,7)	82,8 (±30,8)	83,7 (±31,8)	0,179		68,3 (±17,6)	70,9 (±19,5)	72,3 (±18,5)	0,101
Feminino	65,9 (±24,3)	69,6 (±26,9)	70,0 (±24,7)	0,061		60,9 (±42,2)	64,4 (±41,6)	64,7 (±41,3)	0,514		56,1 (±12,6)	58,4 (±18,6)	61,3 (±19,4)	**0,009**
**p-valor** ^†^	**0,001**	**0,006**	**0,002**			**0,009**	**0,003**	**0,001**			**<0,001**	**<0,001**	**<0,001**	
**Raça/Cor**														
Branca	65,1 (±28,5)	71,9 (±26,5)	72,1 (±23,9)	**0,004**		64,9 (±41,0)	69,5 (±36,3)	71,3 (±38,0)	0,686		57,6 (±20,2)	61,8 (±20,4)	62,8 (±21,0)	**0,031**
Preta	72,0 (±26,4)	69,0 (±26,6)	72,0 (±28,4)	0,932		71,4 (±39,8)	61,9 (±43,8)	66,6 (±44,7)	0,907		62,8 (±20,0)	63,0 (±20,6)	64,1 (±18,8)	0,914
Parda	73,6 (±20,6)	76,7 (±23,7)	77,1 (±23,1)	0,065		67,9 (±39,6)	75,5 (±38,5)	74,5 (±38,0)	0,067		62,8 (±17,5)	64,7 (±19,6)	67,9 (±19,3)	**0,003**
**p-valor** ^§^	0,283	0,340	0,423			0,707	0,144	0,781			0,317	0,662	0,354	
**2ª camada – Estilo de vida dos indivíduos**														
**Atividade física/semana**														
0 a 3 dias	70,5 (±24,8)	73,1 (±25,1)	75,0 (±24,4)	**0,015**		66,2 (±40,2)	71,9 (±38,6)	73,7 (±38,1)	**0,036**		60,6 (±19,8)	62,8 (±20,6)	65,6 (±19,9)	**0,003**
4 a 7 dias	71,6 (±22,0)	79,4 (±24,4)	74,6 (±22,9)	**0,016**		72,5 (±38,9)	72,5 (±38,8)	67,6 (±41,4)	0,617		63,2 (±13,7)	66,9 (±16,5)	66,8 (±19,7)	0,417
**p-valor** ^†^	0,952	0,144	0,844			0,328	0,957	0,415			0,452	0,249	0,721	
**Consumo de frutas e de verduras/semana**														
0 a 3 dias	73,2 (±23,7)	75,5 (±23,3)	76,7 (±23,3)	0,062		70,2 (±38,3)	71,6 (±38,4)	77,3 (±34,9)	0,094		60,7 (±19,2)	63,5 (±20,7)	66,0 (±19,7)	**0,014**
4 a 7 dias	67,9 (±24,6)	72,9 (±26,9)	72,9 (±24,9)	**0,018**		64,3 (±41,7)	72,5 (±38,8)	76,4 (±42,0)	0,118		61,6 (±18,4)	63,6 (±19,1)	65,7 (±20,0)	0,075
**p-valor** ^†^	0,131	0,658	0,299			0,443	0,800	0,152			0,731	0,964	0,986	
**Consumo de frituras e industrializados/semana**														
0 a 3 dias	71,4 (±23,6)	75,4 (±24,2)	77,3 (±23,0)	**<0,001**		67,6 (±39,8)	71,2 (±38,7)	75,8 (±37,4)	0,073		61,3 (±17,9)	63,7 (±20,0)	66,7 (±19,5)	**0,001**
4 a 7 dias	68,7 (±25,9)	71,0 (±27,1)	68,2 (±25,7)	0,239		66,6 (±40,6)	74,3 (±38,4)	63,8 (±41,1)	0,032		60,6 (±21,1)	63,2 (±19,7)	63,5 (±20,6)	0,340
**p-valor** ^†^	0,582	0,573	**0,025**			0,870	0,573	0,057			0,900	0,860	0,423	
**Exposição ao sol/semana**														
0 a 3 dias	72,9 (±23,1)	77,2 (±23,3)	78,4 (±23,1)	**0,003**		71,5 (±37,6)	75,8 (±37,6)	77,6 (±34,8)	0,180		61,1 (±18,4)	63,9 (±20,1)	68,0 (±18,4)	**0,002**
4 a 7 dias	67,3 (±25,7)	69,6 (±26,9)	69,4 (±24,6)	0,212		60,9 (±42,8)	66,1 (±39,5)	64,7 (±43,1)	0,531		61,1 (±19,5)	63,0 (±19,6)	62,6 (±21,5)	0,312
**p-valor** ^†^	0,164	0,061	**0,010**			0,134	0,050	0,069			0,841	0,798	0,140	
**Dificuldade para dormir**														
Sim	65,0 (±25,5)	69,9 (±25,3)	71,3 (±24,4)	**0,004**		59,5 (±41,8)	63,9 (±41,6)	66,3 (±42,4)	0,290		55,5 (±19,0)	59,4 (±20,7)	62,1 (±20,9)	**<0,001**
Não	77,8 (±20,5)	79,6 (±23,8)	79,3 (±23,0)	0,239		77,0 (±35,4)	82,0 (±31,7)	80,4 (±32,1)	0,345		68,1 (±16,0)	68,7 (±17,7)	70,6 (±17,4)	0,575
**p-valor** ^†^	**0,001**	**0,006**	**0,016**			**0,004**	**0,002**	**0,038**			**<0,001**	**0,002**	**0,005**	
**Tabagismo**														
Sim	78,1 (±19,7)	76,5 (±24,4)	79,6 (±25,8)	0,846		91,6 (±15,4)	87,5 (±17,2)	87,5 (±24,8)	0,819		61,5 (±11,6)	64,5 (±20,1)	67,5 (±18,3)	0,670
Não	70,3 (±24,4)	74,1 (±25,1)	74,7 (±24,0)	**0,001**		66,2 (±40,4)	71,3 (±39,1)	71,9 (±39,1)	0,082		61,1 (±19,1)	63,5 (±19,9)	65,8 (±19,9)	**0,002**
**p-valor** ^†^	0,449	0,808	0,533			0,104	0,455	0,313			0,964	0,958	0,911	
**Etilismo**														
Sim	73,8 (±23,2)	75,0 (±25,7)	78,3 (±23,4)	0,117		68,7 (±39,7)	75,5 (±37,6)	77,2 (±36,8)	0,069		61,8 (±16,7)	63,8 (±18,8)	67,0 (±19,3)	0,096
Não	68,2 (±24,8)	73,7 (±24,5)	72,2 (±24,3)	**0,005**		66,3 (±40,3)	69,3 (±39,2)	69,0 (±39,9)	0,707		60,6 (±20,3)	63,4 (±20,8)	65,0 (±20,2)	**0,011**
**p-valor** ^†^	0,129	0,639	0,090			0,702	0,261	0,156			0,836	0,899	0,521	
**3ª camada – Redes sociais e comunitárias**														
**Quantidade de pessoas que reside**														
Mora só ou uma	79,1 (±15,3)	61,1 (±26,1)	72,2 (±23,1)	0,355		59,2 (±49,3)	44,4 (±44,0)	85,1 (±29,3)	0,115		58,2 (±19,7)	62,6 (±23,1)	71,5 (±19,1)	**0,008**
≥ 2	70,2 (±24,5)	75,0 (±24,8)	75,0 (±24,1)	**<0,001**		67,8 (±39,5)	73,5 (±37,8)	71,9 (±39,1)	0,116		61,3 (±18,8)	63,6 (±19,8)	65,6 (±19,8)	**0,008**
**p-valor** ^†^	0,391	0,111	0,597			0,718	**0,048**	0,288			0,506	0,869	0,391	
**Reside com pessoas do grupo de risco**														
Sim	70,8 (±24,2)	75,4 (±24,2)	76,3 (±22,6)	**0,005**		67,4 (±40,8)	71,8 (±38,0)	74,4 (±39,3)	0,160		60,5 (±17,6)	61,1 (±19,1)	64,7 (±18,4)	0,131
Não	70,6 (±24,4)	73,1 (±25,9)	73,4 (±25,4)	0,158		67,4 (±39,2)	72,2 (±39,3)	70,7 (±38,2)	0,397		61,7 (±19,9)	66,1 (±20,5)	67,1 (±21,2)	**0,004**
**p-valor** ^†^	0,973	0,698	0,608			0,898	0,761	0,394			0,742	0,132	0,295	
**4ª camada – Condições de vida**														
**Abastecimento de água**														
Rede de distribuição	70,6 (±24,4)	74,5 (±25,2)	75,6 (±23,9)	**<0,001**		67,6 (±39,8)	72,8 (±38,1)	74,2 (±38,0)	0,081		61,1 (±19,0)	64,1 (±19,8)	66,2 (±19,9)	**<0,001**
Outro	71,5 (±21,7)	70,4 (±23,2)	63,6 (±24,6)	0,200		63,6 (±43,3)	60,6 (±44,2)	48,4 (±43,1)	0,174		61,8 (±15,4)	54,9 (±19,1)	60,3 (±17,9)	0,417
**p-valor** ^†^	0,976	0,445	0,083			0,672	0,315	**0,020**			0,969	0,174	0,282	
**Escoadouro de esgoto**														
Rede de esgoto	69,4 (±24,0)	73,1 (±24,7)	75,2 (±23,2)	**0,003**		65,8 (±39,4)	72,2 (±38,7)	73,0 (±37,2)	0,103		59,8 (±18,6)	61,4 (±19,1)	65,4 (±19,0)	**0,002**
Outro	73,3 (±24,6)	76,6 (±25,6)	74,3 (±25,8)	0,190		70,6 (±41,0)	71,7 (±38,5)	71,7 (±41,8)	0,837		63,7 (±19,0)	68,0 (±20,9)	66,7 (±21,5)	0,100
**p-valor** ^†^	0,269	0,241	0,995			0,239	0,766	0,910			0,221	**0,029**	0,652	
**5ª Camada – Condições socioeconômicas, culturais e ambientais gerais**														
**Renda familiar**														
< 3 salários mínimos	70,5 (±24,4)	74,2 (±25,2)	73,9 (±25,1)	**0,007**		69,1 (±39,3)	72,8 (±37,7)	73,2 (±38,9)	0,293		61,2 (±19,8)	64,1 (±20,4)	65,3 (±21,0)	**0,040**
> 3 salários mínimos	71,0 (±24,1)	74,3 (±24,9)	76,3 (±22,4)	0,117		64,8 (±41,0)	70,8 (±39,9)	71,7 (±38,6)	0,404		61,0 (±17,2)	62,7 (±19,1)	66,7 (±17,9)	**0,025**
**p-valor** ^†^	0,918	0,939	0,718			0,551	0,818	0,637			0,984	0,637	0,776	

Legenda: *Teste de Frideman/

^§^Kruskal-Wallis/

^†^Mann-Whitney.

Na primeira camada, participantes com 40 a 59 anos evoluíram significativamente entre o 2º e 6º mês em capacidade funcional (p = 0,033), dor (p = 0,042), vitalidade (p = 0,009) e aspectos sociais (p = 0,012). Já entre adultos jovens (20 a 39 anos) e idosos (60 a 75 anos), a melhoria concentrou-se na saúde mental, com maior variação entre o 2º e 6º mês (p = 0,009 e p = 0,045, respectivamente). Comparativamente, participantes de 20 a 39 anos mantiveram escores de capacidade funcional superior aos de 40 a 59 anos e 60 a 75 anos no 2º mês (p = 0,016 e p = 0,021, respectivamente), sustentando essa diferença sobre os idosos no 4º (p = 0,026) e 6º mês (p = 0,013). No 6º mês, a idade também influenciou a dor, sendo menor a percepção de dor (maiores escores) em jovens e meia-idade comparados aos idosos (p = 0,004 e p = 0,010, respectivamente) (Tabelas 1, 2 e 3).

Quanto ao sexo, homens apresentaram melhor recuperação em estado geral de saúde (2º e 4º mês, p = 0,029) e aspectos sociais (2º e 6º mês, p = 0,040). As mulheres evoluíram em dor (2º e 4º mês, p = 0,038), vitalidade (4º e 6º mês, p = 0,035) e saúde mental (2º e 6º mês, p = 0,005). Na comparação entre grupos, os homens apresentaram médias superiores em praticamente todos os domínios. Diferenças significativas mantiveram-se ao longo dos três meses para capacidade funcional, vitalidade e saúde mental (p < 0,001 em todos). Para limitações por aspectos físicos, dor, estado geral de saúde, aspectos sociais e aspectos emocionais, a superioridade masculina foi estatisticamente significativa na maioria dos pontos avaliados com valores p<0,05 (Tabelas 1, 2 e 3).

A raça/cor modulou o tempo de recuperação: brancos melhoraram aspectos sociais e saúde mental precocemente (2º e 4º mês, p = 0,025 e p = 0,035, respectivamente) e tardiamente (4º e 6º mês para aspectos sociais, p = 0,011). Já os pardos concentraram sua evolução entre o 2º e 6º mês em estado geral de saúde (p = 0,022) e saúde mental (p = 0,002). No 6º mês, pardos superaram brancos no domínio vitalidade (p = 0,014) (Tabelas 2 e 3).

Na segunda camada, a frequência de atividade física influenciou o padrão de melhora. Sedentários ou pouco ativos (0 a 3 dias/semana) evoluíram entre o 2º e 6º mês em limitações por aspectos físicos (p = 0,019), vitalidade (p = 0,010), aspectos sociais (p = 0,022) e saúde mental (p = 0,002). Já os mais ativos (4 a 7 dias) apresentaram ganho pontual em aspectos sociais (2º e 4º mês, p = 0,018), mas sustentaram médias consistentemente superiores aos pouco ativos em capacidade funcional no 2º (p = 0,010), 4º (p = 0,010) e 6º mês (p = 0,042); limitações por aspectos físicos no 2º mês (p = 0,009); e dor no 2º (p = 0,028) e 4º mês (p = 0,021) (Tabelas 1, 2 e 3).

A alimentação e exposição solar também impactaram a trajetória. Baixa ingestão de frutas e verduras (0 a 3 dias/semana) mostrou melhora na saúde mental, especialmente entre o 2º e 6º mês (p = 0,008). Já entre os que consumiam com maior frequência (4 a 7 dias), houve melhora nos aspectos sociais com diferença significativa entre o 2º e 4º mês (p = 0,046). A menor ingestão de alimentos fritos e industrializados associou-se à melhora entre o 2º e 6º mês em capacidade funcional (p = 0,026), vitalidade (p = 0,005) e saúde mental (p < 0,001). O domínio aspectos sociais apresentou diferença entre o 2º e 4º mês (p = 0,036). Aqueles que ingeriam frituras e industrializados de 0 a 3 dias por semana apresentaram melhores escores no domínio aspectos sociais no 6º mês (p = 0,025) (Tabelas 1, 2 e 3).

Participantes com menor exposição solar (0 a 3 dias/semana) apresentaram melhoras observadas entre o 2º e 6º mês para vitalidade (p = 0,005), aspectos sociais (p = 0,010) e saúde mental (p = 0,001), e entre o 2º e 4º mês para estado geral de saúde (p = 0,030) e aspectos sociais (p = 0,046). Aqueles que se expunham ao sol de 0 a 3 dias por semana, também apresentaram maiores médias em aspectos sociais no 6º mês (p = 0,010) (Tabelas 2 e 3).

A dificuldade para dormir apresentou diferenças entre o 2º e 4º mês nos domínios dor (p = 0,036), aspectos sociais (p = 0,009) e saúde mental (p < 0,001), além de diferença entre o 2º e 6º mês para estado geral de saúde (p = 0,030). Entre não tabagistas, observou-se diferença nos seguintes domínios: dor (2º e 4º mês, p = 0,029); vitalidade (2º e 6º mês, p = 0,022); aspectos sociais (2º e 4º mês, p = 0,014 e 4º e 6º mês, p = 0,008); e saúde mental (o 2º e 6º mês, p = 0,001) (Tabelas 2 e 3).

Indivíduos sem consumo de álcool apresentaram evolução positiva em dor, com diferenças significativas entre o 2º e 4º mês nos domínios dor (p = 0,012) e aspectos sociais (p = 0,012). Entre o 2º e 6º mês as diferenças foram nos domínios vitalidade (p = 0,010), aspectos sociais (p = 0,031) e saúde mental (p = 0,006) (Tabelas 2 e 3).

Na terceira camada, o suporte social foi relevante. Residir sozinho ou com uma pessoa apresentou melhora significativa somente na saúde mental (2º e 6º mês, p = 0,007). Aqueles que residiam com duas ou mais pessoas demonstraram melhorias em vitalidade (2º e 6º mês, p = 0,016) e saúde mental (2º e 6º mês, p = 0,005). No domínio aspectos sociais a diferença ocorreu entre o 2º e 4º mês (p = 0,005) (Tabelas 2 e 3).

A convivência com pessoas do grupo de risco esteve associada a melhorias entre o 4º e 6º mês para vitalidade (p = 0,021) e entre o 2º e 4º mês para aspectos sociais (p = 0,031). Entre os que não residiam com pessoas do grupo de risco, observaram-se diferenças estatisticamente significativas entre o 2º e 4º mês para os domínios vitalidade (p = 0,013) e saúde mental (p = 0,033), bem como entre o 2º e 4º mês (p = 0,039) e entre o 4º e 6º mês (p = 0,036) para o domínio estado geral de saúde (Tabelas 2 e 3).

Na quarta camada, condições de vida, participantes que residiam em locais com abastecimento de água por rede pública apresentaram diferenças evidenciadas nos domínios de vitalidade entre o 2º e 6º mês (p = 0,006), aspectos sociais entre o 2º e 4º mês (p = 0,010) e saúde mental entre o 2º e 4º mês (p = 0,022). Aqueles que dispunham de abastecimento de água por rede pública demonstraram melhores médias no domínio aspectos emocionais no 6º mês (p = 0,020) (Tabelas 2 e 3).

Em relação ao tipo de escoamento sanitário, os indivíduos residentes em áreas com rede de esgotamento sanitário apresentaram melhora significativas em vitalidade entre o 2º e 6º mês (p = 0,012), aspectos sociais entre o 2º e 4º mês (p = 0,045) e saúde mental entre o 4º e 6º mês (p = 0,033) (Tabelas 2 e 3).

Na quinta camada, participantes com renda familiar mensal inferior a três salários mínimos apresentaram melhoria com diferenças significativas em aspectos sociais (o 2º e 4º mês, p = 0,022) e saúde mental (2º e 6º mês, p = 0,022). Para os participantes com renda superior a três salários mínimos, as diferenças ocorreram em vitalidade (4º e 6º mês, p = 0,041) e saúde mental (2º e 6º mês, p = 0,012) (Tabelas 2 e 3).

## DISCUSSÃO

Os resultados evidenciam que os DSS exercem influência sobre a QV de pacientes diagnosticados com COVID-19 ao longo do tempo, confirmando que a trajetória de melhora não depende apenas da resolução clínica, mas das condições estruturais e comportamentais. A predominância de adultos jovens alinha-se à mudança no perfil epidemiológico da COVID-19^(15)^, em contraste com a média de idade mais elevada observada no início da pandemia^(16)^, o que pode ter favorecido a recuperação funcional observada, em contraste com a pior QV relatada em idosos após a infecção^(17)^.

Quanto ao sexo, a maior proporção de mulheres reflete o engajamento feminino em práticas de cuidado com a saúde e testagem para COVID-19^(18)^. Contudo, os homens apresentaram melhor recuperação em aspectos físicos e sociais, enquanto mulheres obtiveram melhores resultados em saúde mental. Essa disparidade sugere diferenças de enfrentamento e impacto psicossocial da pandemia entre os gêneros^(19)^.

Na raça/cor, a predominância de pessoas autodeclaradas pardas reflete a composição demográfica brasileira^(20)^ e achados de estudos anteriores com populações acometidas pela COVID-19^(20,21,22)^. A distinção na recuperação entre brancos (melhores em saúde mental) e pardos (aspectos físicos) reforça o peso das desigualdades estruturais e do estresse social enfrentado por grupos racializados no Brasil^(22)^.

O estilo de vida emergiu como fator central. O alto sedentarismo reflete os impactos do isolamento social durante a pandemia^(23)^, mas a prática regular de atividade física confirmou-se como protetora para a vitalidade e saúde mental^(24)^. Padrões semelhantes foram notados na alimentação e sono: o consumo de alimentos saudáveis associou-se a melhores desfechos psicossociais^(25)^, enquanto distúrbios do sono e baixa exposição solar associaram-se a piores indicadores em diversos domínios, evidenciando a relevância da luz solar para a síntese de vitamina D e o equilíbrio emocional, e o impacto do confinamento no ritmo circadiano e bem-estar^(26)^. Esses achados reforçam o papel dos comportamentos relacionados ao estilo de vida, camada intermediária do modelo de Dahlgren e Whitehead, na modulação da qualidade de vida durante o período pós-COVID-19.

O tabagismo apresentou baixa prevalência, corroborando estudos que apontam uma maior preocupação com doenças respiratórias durante a crise sanitária^(27)^. A ausência de tabagismo e o não consumo de álcool atuaram como fatores de proteção para a QV geral, contrariando tendências de aumento do consumo durante a pandemia^(23,28)^.

No âmbito das redes sociais e comunitárias, a coabitação com maior número de pessoas ou grupos de risco impactou negativamente a vitalidade e dor, reflexo da dificuldade de isolamento e sobrecarga de cuidados em ambientes^(20,29)^. Nas condições de moradia, o acesso a saneamento básico (água e esgoto) mostrou-se fundamental para a recuperação em quase todos os domínios de QV^(30)^. A renda familiar, majoritariamente baixa, revela um importante marcador de vulnerabilidade que pode aumentar o risco de exposição e dificultar o acesso a recursos essenciais^(31)^. No entanto, este estudo revelou que famílias com até três salários mínimos apresentaram melhores resultados em aspectos físicos e sociais, enquanto as de renda mais alta destacaram-se apenas no domínio vitalidade. Esta nuance sugere que a renda, embora um DSS crítico, pode ter interações complexas com outros fatores de proteção ou vulnerabilidade pós-COVID-19.

A interpretação dos achados à luz do modelo de Dahlgren e Whitehead^(6)^ permite compreender que os DSS atuam de forma interdependente na recuperação da QV após a COVID-19^(32)^. Fatores individuais, como idade, sexo e raça/cor, interagem com comportamentos relacionados ao estilo de vida, redes sociais e comunitárias, bem como com condições de moradia e renda, influenciando de maneira distinta os domínios físicos e mentais da QV ao longo do seguimento. Nesse sentido, observou-se que estilo de vida, condições de moradia, renda e redes de apoio modularam a trajetória de recuperação, evidenciando que indivíduos expostos a contextos sociais mais favoráveis apresentaram melhor evolução em determinados domínios dos SF-36. Esses achados reforçam que a recuperação pós-COVID-19 não ocorre de forma homogênea, sendo permeada por desigualdades sociais que influenciam o processo de reabilitação.

A evolução positiva dos escores do SF-36 ao longo dos seis meses é consistente com estudos internacionais^(14,33)^, indicando uma restauração progressiva da saúde. O acompanhamento por um período de seis meses foi essencial para determinar os impactos dos DSS no decorrer do tempo, assim como observar a tendência de evolução da QV dos indivíduos acometidos pela COVID-19. A influência dos DSS reforça a necessidade de estratégias intersetoriais que trabalhem com foco direcionado principalmente aos DSS que exercem impactos diretos na QV dos indivíduos. A Enfermagem, na Atenção Primária, deve liderar o planejamento de cuidados que integrem a promoção de hábitos saudáveis e o fortalecimento de redes de apoio, considerando as iniquidades regionais para garantir uma reabilitação equitativa^(12)^.

É importante reconhecer as limitações deste estudo. A restrição geográfica aos estados do Ceará e Maranhão limita a generalização dos achados a nível nacional. Além disso, não foram controlados possíveis fatores intervenientes ao longo do seguimento, como reinfecção por COVID-19, agravamento do quadro clínico, acesso a serviços de reabilitação ou mudanças nas condições de vida dos participantes, que poderiam influenciar os escores de qualidade de vida observados. A descontinuidade de participantes ao longo do seguimento (de 391 para 179 participantes), embora prevista no cálculo amostral, pode ter atenuado a força de algumas associações. Apesar disso, os achados oferecem subsídios relevantes para a gestão pública e a prática clínica no contexto pós-pandêmico.

## CONCLUSÃO

Conclui-se que a QV de indivíduos em condição pós-COVID-19 apresentou melhora progressiva e estatisticamente significante ao longo do tempo, sendo esta trajetória intrinsecamente influenciada pelos DSS. Fatores relacionados ao estilo de vida, condições de moradia, renda e redes de apoio modularam a recuperação dos domínios físicos e mentais, evidenciando que o processo de reabilitação não ocorre de forma homogênea, mas é permeado por desigualdades sociais que impactam a evolução da QV.

Estes achados reforçam a necessidade de que a Enfermagem e a gestão pública implementem estratégias longitudinais que transcendam o manejo clínico da condição pós-COVID-19. Nesse sentido, destacam-se ações intersetoriais voltadas à promoção de hábitos saudáveis, incentivo à prática de atividade física, educação em saúde sobre alimentação e sono, fortalecimento das redes de apoio social, bem como políticas públicas voltadas à melhoria das condições de moradia, saneamento básico e acesso equitativo aos serviços de saúde e reabilitação.

Como contribuição científica, este estudo avança ao analisar longitudinalmente a influência dos DSS na QV pós-COVID-19, evidenciando como diferentes camadas do modelo de Dahlgren e Whitehead interagem no processo de recuperação. Esses resultados contribuem para ampliar a compreensão das desigualdades no contexto pós-pandêmico e oferecer subsídios para o planejamento de intervenções de Enfermagem na Atenção Primária à Saúde, orientadas pela equidade e pela promoção da saúde. Recomenda-se a replicação do estudo em outros contextos para ampliar a compreensão do impacto da pandemia na população brasileira e subsidiar políticas de saúde orientadas pela equidade.

## Data Availability

Todo o conjunto de dados que dá suporte aos resultados deste estudo foi publicado no próprio artigo.

# Translation: Determinantes sociales de la salud y la calidad de vida después de la COVID-19: un estudio longitudinal

**DOI:** 10.1590/1980-220X-REEUSP-2025-0565es

**Authors:** Sabrina de Souza Gurgel Florencio, Francisca Elisângela Teixeira Lima, Glaubervania Alves Lima, Maria Gabriela Miranda Fontenele, Lívia Maia Pascoal, Lilia Jannet Saldarriaga Sandoval, Paulo César de Almeida, Gilvan Ferreira Felipe, Maria Williany Silva Ventura, Brena Shellem Bessa de Oliveira, Lorena Pinheiro Barbosa

**Affiliations:** 1 Universidade Federal do Ceará, Fortaleza, CE, Brasil.; 2 Universidade Federal do Maranhão, Imperatriz, MA, Brasil.; 3 Universidad Nacional de Tumbes, Tumbes, Peru.; 4 Universidade Estadual do Ceará, Fortaleza, CE, Brasil.; 5 Universidade da Integração Internacional da Lusofonia Afro-Brasileira, Redenção, CE, Brasil.

**Keywords:** COVID-19, Determinantes Sociales de la Salud, Calidad de Vida, Enfermería, Salud Pública

## Abstract

**Objetivo:**

Verificar la asociación entre los determinantes sociales de la salud y los dominios de la calidad de vida en personas en situación posterior a la COVID-19.

**Métodos:**

Se realizó un estudio longitudinal con 179 personas de los estados de Ceará y Maranhão, a quienes se les dio seguimiento durante seis meses después de un diagnóstico confirmado de COVID-19. La recopilación de datos se realizó a través de enlace de *software* de seguimiento de pacientes con COVID-19, enviados mensualmente a través de una aplicación de mensajería móvil. Se consideran variables relacionadas con los determinantes sociales de la salud y los dominios del cuestionario SF-36 para evaluar la calidad de vida. Los datos se analizaron utilizando estadística descriptiva e inferencial.

**Resultados:**

Las mujeres mostraron mejoras en el ámbito del dolor entre el segundo y el cuarto mes (p = 0,038), en la vitalidad entre el cuarto y el sexto mes (p = 0,035) y en la salud mental entre el segundo y el sexto mes (p = 0,005). Los hombres mostraron una mejor recuperación en el estado de salud general a los 2 y 4 meses (p = 0,029) y en los aspectos sociales a los 2 y 6 meses (p = 0,040).

**Conclusión:**

Durante el período de seguimiento se observó una mejora progresiva en la calidad de vida, con diferencias entre hombres y mujeres en las áreas de dolor, vitalidad, salud mental, estado de salud general y aspectos sociales.

## INTRODUCCIÓN

La pandemia de COVID-19 desencadenó una crisis sanitaria global cuyos impactos superaron la fase aguda de la infección, generando repercusiones prolongadas en las condiciones físicas, emocionales y sociales de las personas afectadas, configurando la condición pos-COVID-19^(1,2)^. Este escenario afectó directamente la calidad de vida (QV) de la población, entendida como la percepción del individuo sobre su posición en la vida, en el contexto cultural y de valores en el que se inserta^(3)^. Durante la pandemia, la QV se volvió vulnerable a los cambios en los hábitos cotidianos, las condiciones de trabajo, la vivienda, la alimentación y el acceso a los servicios de salud^(4)^.

Dichos elementos forman parte de los Determinantes Sociales de la Salud (DSS), factores sociales, económicos, culturales, étnico-raciales, psicológicos y conductuales que influyen en el riesgo de enfermedad y mortalidad en las poblaciones^(5)^. Para comprender esta interacción, este estudio adopta el modelo de Dahlgren y Whitehead^(6)^, que organiza los DSS en capas concéntricas, desde factores individuales, estilos de vida, redes sociales y comunitarias, hasta condiciones de vida y de trabajo y, finalmente, los macrodeterminantes socioeconómicos. Esta estructura pone de manifiesto la compleja interacción entre el individuo y su contexto social y ambiental en la determinación de la salud.

Al actuar sobre cada una de estas capas, la pandemia de COVID-19 funcionó como un potente intensificador de las desigualdades estructurales ya descritas en el modelo de Dahlgren y Whitehead, haciendo aún más evidentes las vulnerabilidades preexistentes. En este contexto, los grupos con un nivel socioeconómico más bajo, saneamiento inadecuado, vivienda precaria y acceso limitado a una alimentación saludable y a los servicios de salud se vieron afectados de manera desproporcionada^(7)^. Dichas desigualdades aumentaron el riesgo de infección y mortalidad, y comprometieron la CV de los sobrevivientes. Aunque la literatura ha investigado la CV post-COVID-19^(8,9)^, aún son limitados los estudios que analizan la influencia de los DSS en la evolución de la CV a lo largo del tiempo, especialmente en investigaciones longitudinales realizadas en estados del noreste de Brasil como Ceará y Maranhão, caracterizados por desigualdades socioeconómicas históricamente más pronunciadas.

En Brasil, la pandemia puso de manifiesto brechas históricas en el acceso a los derechos sociales básicos. La implementación de medidas de emergencia, como el Programa de Ayuda de Emergencia (PAE), un beneficio financiero temporal creado por el gobierno federal para reducir los impactos económicos de la pandemia entre las poblaciones socialmente vulnerables, aunque necesaria, también expuso limitaciones estructurales en la capacidad de respuesta y protección social del Estado^(10)^. En este escenario, la evaluación de los DSS y su influencia en la CV post-COVID-19 es fundamental para identificar a los grupos más afectados y respaldar políticas públicas más equitativas y eficaces.

En este contexto, es necesario evaluar cómo los determinantes sociales de la salud influyen en la calidad de vida de las personas tras la infección. La evaluación de la CV, mediante instrumentos sensibles a los cambios temporales como el *Short-Form Health Survey* (SF-36), resulta fundamental para identificar vulnerabilidades persistentes^(11)^. El diseño longitudinal constituye una diferencia metodológica relevante, ya que permite seguir la trayectoria de recuperación a lo largo del tiempo, captando variaciones en los dominios de la CV y la influencia de los DSS en diferentes momentos del período pos-COVID-19, algo que los estudios transversales no logran evidenciar. Por lo tanto, es necesario comprender qué DSS influyen en la CV de las personas en condición pos-COVID-19. Esta comprensión contribuye a la producción de conocimiento científico y a la planificación de acciones en salud que apunten a la prevención de afecciones, la rehabilitación funcional y la promoción de la salud desde una perspectiva integral y equitativa^(12)^.

Por lo tanto, el presente estudio tiene como objetivo verificar la asociación entre los DSS y los dominios de CV en personas en condición pos-COVID-19. Los resultados pueden servir de apoyo a enfermeros y gestores de salud en la elaboración de estrategias de atención longitudinal que tengan en cuenta las desigualdades regionales, orientando las intervenciones hacia la rehabilitación funcional, la promoción de la salud mental y el fortalecimiento del apoyo social en el contexto pospandémico.

## MÉTODO

### Tipo de Estudio

Se trata de un estudio longitudinal, analítico y cuantitativo, realizado en los estados de Ceará y Maranhão, regiones que presentaron una elevada incidencia de COVID-19 durante la pandemia. El seguimiento se llevó a cabo durante seis meses, con el envío mensual de formularios de monitoreo y la aplicación del SF-36 en el 2.º, 4.º y 6.º mes, para individuos diagnosticados con COVID-19.

### Protocolo del Estudio

La población de referencia estuvo compuesta por 573 555 adultos y adultos mayores (≥ 20 años), con síntomas gripales notificados en el Sistema de Información de Vigilancia de la Gripe (SIVEP-GRIPE) y/o en el e-SUS Notifica, de los cuales 367 265 eran de Ceará y 206 290 de Maranhão. Teniendo en cuenta un error muestral del 7 % y un intervalo de confianza del 95 %, se estimó una muestra mínima de 179 participantes.

Se incluyeron en la muestra individuos con diagnóstico confirmado de COVID-19 y número de teléfono celular con acceso a internet registrado en los mencionados sistemas de información de las Secretarías de Salud de cada estado. Se excluyó a aquellos con limitación cognitiva autoinformada o que no respondieron tras tres intentos de contacto. De las 391 personas elegibles que respondieron al primer formulario, hubo 212 desistimientos, motivados principalmente por falta de respuesta tras nuevos intentos de contacto (n = 168), negativa a continuar participando (n = 28) y cambio o desactivación del número de teléfono (n = 16), lo que dio como resultado una muestra final de 179 participantes, 95 de Ceará y 84 de Maranhão.

Para la recolección de datos, se utilizó el *software* de seguimiento de pacientes con COVID-19, desarrollado y validado en Brasil^(13)^, el cual contiene dos instrumentos: un formulario de caracterización sociodemográfica y estilo de vida, que abarca los DSS (incluidas variables de edad, sexo, raza/color, actividad física, hábitos alimentarios, exposición solar, calidad del sueño, tabaquismo, alcoholismo, composición del hogar, convivencia con grupos de riesgo, saneamiento básico e ingresos familiares, organizadas según los estratos del modelo de Dahlgren y Whitehead); y el *Short-Form Health Survey* (SF-36), traducido y validado para Brasil, compuesto por 36 ítems distribuidos en ocho dominios (capacidad funcional, aspectos físicos, dolor, estado general de salud, vitalidad, aspectos sociales, aspectos emocionales y salud mental)^(11)^.

La recolección se llevó a cabo entre junio de 2021 y junio de 2022. Inicialmente, se obtuvieron datos de los sistemas SIVEP-GRIPE y e-SUS Notifica, que registran casos sospechosos y confirmados de COVID-19 en el país.

Las fichas de notificación fueron completadas por profesionales de la salud y consolidadas por las Vigilancias Epidemiológicas de las Secretarías Estatales de Salud (SESA) de Ceará y Maranhão.

Tras la autorización de las SESA, los datos se exportaron al software de monitoreo, que generaba un enlace individual para cada participante. Este enlace, acompañado de una invitación a participar, un Término de Consentimiento Libre e Informado y un formulario mensual de seguimiento, se envió por WhatsApp, una aplicación de mensajería ampliamente utilizada en Brasil, lo que pudo haber favorecido la inclusión de participantes con mayor acceso a Internet y alfabetización digital, lo que constituye una limitación del método de reclutamiento. El SF-36 se aplicó en el segundo, cuarto y sexto mes de seguimiento. Estos intervalos se definieron basándose en la literatura sobre la condición pos-COVID-19, que señala los primeros seis meses como el período de mayor variabilidad en la recuperación clínica y funcional, con evaluaciones bimestrales para captar cambios progresivos en los dominios de la CV^(2,14)^. El plazo de respuesta fue de tres días.

### Análisis de los Datos

Los datos se analizaron en el programa *Statistical Package for the Social Sciences* (SPSS), versión 20.0, licencia 10101131007. Las variables se describieron mediante frecuencias absolutas, relativas, medias y desviaciones estándar. La normalidad de los datos se evaluó mediante la prueba de *Kolmogorov-Smirnov*.

Ante la distribución no normal, se aplicó la prueba de Friedman, seguida de la prueba de Conover para comparaciones múltiples entre el 2.º, 4.º y 6.º mes, con el fin de verificar la evolución de los dominios del SF-36 y su asociación con los determinantes sociales de la salud. Para la comparación entre grupos independientes, se utilizaron las pruebas de Kruskal-Wallis y Mann-Whitney. En las variables con diferencias significativas según el Kruskal-Wallis, se realizó una prueba post hoc de Dunn con corrección de Bonferroni. El nivel de significación adoptado fue p < 0,05.

### Aspectos Éticos

El estudio fue aprobado por el Comité de Ética en Investigación, bajo el dictamen n.º 4.278.495/2020 y el identificador CAAE 36193820.1.0000.5054.

## RESULTADOS

Participaron en el estudio 179 individuos, predominantemente mujeres (58,7 %), con una edad media de 37,4 ± 12,6 años y que se autodefinieron como pardos (56,4 %). Se observó una baja adherencia a la actividad física regular (81,0 %), un consumo insuficiente de frutas y verduras (52,5 %), una alta ingesta de alimentos fritos y procesados (73,2 %), una baja exposición al sol (60,9 %) y dificultades para dormir (55,3 %). El 44,1 % declaró consumir alcohol y el 4,5 % eran fumadores.

La mayoría residía con dos o más personas (95,0 %), convivía con individuos del grupo de riesgo (50,3 %), tenía acceso a la red general de distribución de agua (93,9 %), a la red de alcantarillado (67,0 %) y vivía con un ingreso mensual de hasta tres salarios mínimos (59,8 %) (Tabla 1).

**Tabla 1:** Asociación de los determinantes sociales de la salud de las personas con COVID-19 con las medias de los dominios “Capacidad funcional” y “Limitaciones por aspectos físicos” del instrumento SF-36, comparando el 2.º, 4.ºy 6.ºmes – Fortaleza, CE, Brasil, 2023.

Dominios SF-36	Total			Capacidad funcional media (±DE)					Limitaciones por aspectos físicos			
	n	%		2.º mes	4.º mes	6.º mes	p*		2.º mes	4.º mes	6.º mes	p*
**1.ª camada: edad, sexo y factores hereditarios**												
**Edad**												
20–39	114	63,7		83,4 (±20,4)	83,7 (±19,2)	84,2 (±18,4)	0,905		76,5 (±35,7)	82,2 (±30,9)	84,2 (±27,6)	0,070
40–59	56	31,3		75,8 (±22,8)	76,9 (±23,6)	78,8 (±21,2)	**0,038**		71,4 (±37,9)	73,2 (±38,6)	74,5 (±36,1)	0,645
60–75	9	5,0		66,6 (±25,1)	68,3 (±23,3)	69,4 (±16,2)	0,747		66,6 (±45,0)	75,0 (±39,5)	69,4 (±46,3)	0,819
**valor p** ^§^				**0,008**	**0,034**	**0,027**			0,634	0,432	0,254	
**Sexo**												
Masculino	105	58,7		87,4 (±17,0)	86,6 (±18,5)	88,1 (±17,1)	0,591		80,0 (±34,8)	88,5 (±25,5)	89,8 (±23,0)	**0,038**
Mujeres	74	41,3		75,1 (±23,4)	76,6 (±22,1)	77,4 (±19,9)	0,331		70,4 (±37,7)	72,3 (±37,6)	73,8 (±35,4)	0,467
**valor p** ^†^				**<0,001**	**0,001**	**<0,001**			**0,042**	**0,002**	**0,001**	
**Raza/Color**												
Blanca	57	31,8		79,3 (±26,4)	80,5 (±22,2)	83,0 (±20,2)	0,560		74,5 (±39,1)	76,3 (±33,8)	77,6 (±32,2)	0,672
Negra	21	11,7		80,0 (±21,8)	78,8 (±23,6)	76,1 (±20,9)	0,115		71,4 (±35,6)	70,2 (±40,7)	80,9 (±34,3)	0,353
Parda	101	56,4		80,7 (±18,9)	81,4 (±20,3)	82,3 (±18,7)	0,383		75,0 (±36,0)	82,4 (±32,5)	81,9 (±31,2)	**0,024**
**valor p** ^§^				0,745	0,951	0,307			0,754	0,208	0,549	
**2.ª capa : estilo de vida de los individuos**												
**Actividad física/semana**												
De 0 a 3 días	145	81,0		78,6 (±21,9)	79,2 (±21,6)	80,5 (±19,7)	0,236		71,5 (±37,8)	79,3 (±34,1)	81,5 (±30,7)	**<0,001**
4 a 7 días	34	19,0		86,9 (±20,1)	87,9 (±18,3)	87,3 (±17,7)	0,500		86,7 (±29,6)	77,9 (±34,1)	75,7 (±36,1)	**0,026**
**valor p** ^†^				**0,010**	**0,010**	**0,042**			**0,009**	0,832	0,583	
**Consumo de frutas y verduras/semana**												
De 0 a 3 días	94	52,5		78,3 (±21,6)	79,8 (±20,8)	80,7 (±19,7)	0,179		76,5 (±34,5)	78,9 (±32,4)	81,1 (±29,9)	0,300
4 a 7 días	85	47,5		82,2 (±21,9)	81,9 (±21,7)	83,0 (±19,2)	0,805		72,0 (±39,2)	79,1 (±35,9)	79,7 (±33,9)	0,121
**valor p** ^†^				0,071	0,280	0,391			0,760	0,686	0,810	
**Consumo de fritos y productos procesados/semana**												
De 0 a 3 días	131	73,2		79,3 (±22,5)	81,1 (±21,9)	83,0 (±19,2)	**0,026**		75,0 (±35,7)	79,0 (±33,9)	79,9 (±31,5)	0,082
4 a 7 días	48	26,8		82,5 (±19,5)	80,1 (±19,3)	78,6 (±20,1)	0,136		72,9 (±39,8)	79,1 (±34,7)	81,7 (±32,9)	0,353
**valor p** ^†^				0,420	0,912	0,119			0,818	0,482	0,510	
**Exposición al sol/semana**												
De 0 a 3 días	109	60,9		79,0 (±22,0)	82,1 (±19,5)	82,1 (±18,9)	0,110		78,8 (±32,6)	81,6 (±31,1)	81,8 (±30,2)	0,202
4 a 7 días	70	39,1		82,0 (±21,4)	78,8 (±23,6)	81,3 (±20,4)	0,580		67,5 (±41,7)	75,0 (±38,0)	78,2 (±34,2)	0,198
**valor p** ^†^				0,254	0,636	0,922			0,147	0,334	0,589	
**Dificultad para dormir**												
Sí	99	55,3		77,4 (±22,8)	80,5 (±21,4)	79,9 (±20,7)	0,170		82,1 (±31,3)	85,0 (±29,9)	85,9 (±26,3)	0,133
No	80	44,7		83,6 (±20,0)	81,3 (±21,0)	84,1 (±17,7)	0,591		68,1 (±39,7)	74,2 (±36,5)	76,0 (±35,1)	0,340
**valor p** ^†^				**0,035**	0,748	0,244			**0,019**	**0,022**	0,059	
**Tabaquismo**												
Sí	8	4,5		86,2 (±24,0)	90,6 (±10,5)	87,5 (±18,5)	0,953		100 (±0,0)	90,6 (±26,5)	100 (±0,0)	0,368
No	171	95,5		79,9 (±21,7)	80,4 (±21,5)	81,5 (±19,5)	0,427		73,2 (±37,2)	78,5 (±34,3)	79,5 (±32,2)	**0,041**
**valor p** ^†^				0,167	0,319	0,337			**0,019**	0,224	**0,043**	
**Alcoholismo**												
Sí	79	44,1		83,1 (±20,3)	83,2 (±19,9)	84,3 (±18,0)	0,756		76,8 (±35,0)	82,2 (±30,7)	85,1 (±27,8)	0,164
No	100	55,9		77,9 (±22,7)	79,0 (±22,1)	79,9 (±20,4)	0,160		72,5 (±38,1)	76,5 (±36,3)	76,7 (±34,3)	0,256
**valor p** ^†^				0,136	0,274	0,211			0,461	0,388	0,126	
**3.ª capa: redes sociales y comunitarias**												
**Número de personas que residen**												
Vive sola o con una	9	5,0		75,5 (±23,7)	76,1 (±25,0)	78,8 (±26,1)	0,857		72,2 (±42,2)	72,2 (±44,0)	91,6 (±17,6)	0,779
≥ 2	170	95,0		80,4 (±21,7)	81,1 (±21,0)	82,0 (±19,1)	0,481		74,5 (±36,6)	79,4 (±33,5)	79,8 (±32,3)	**0,043**
**valor p** ^†^				0,471	0,551	0,970			0,819	0,802	0,351	
**Vive con personas del grupo de riesgo**												
Sí	90	50,3		79,4 (±21,6)	80,7 (±20,7)	82,3 (±20,0)	0,819		73,3 (±37,8)	80,2 (±33,6)	80,8 (±32,4)	**0,018**
No	89	49,7		80,9 (±22,0)	80,9 (±21,8)	81,3 (±19,0)	0,114		75,5 (±35,9)	77,8 (±34,6)	80,0 (±31,3)	0,416
**valor p** ^†^				0,525	0,678	0,717			0,687	0,597	0,815	
**4.ª capa – Condiciones de vida**												
**Suministro de agua**												
Red de distribución	168	93,9		81,1 (±20,2)	81,5 (±21,0)	82,3 (±19,1)	0,387		74,1 (±36,9)	80,0 (±33,5)	81,5 (±30,4)	**0,017**
Otros	11	6,1		66,8 (±37,7)	70,4 (±22,6)	74,0 (±24,5)	0,882		79,5 (±35,0)	63,6 (±39,3)	63,6 (±46,5)	0,444
**valor p** ^†^				0,508	0,054	0,276			0,642	0,119	0,212	
**Desagüe de alcantarillado**												
Red de alcantarillado	120	67,0		79,7 (±20,7)	81,5 (±19,6)	81,5 (±19,3)	0,526		73,9 (±36,8)	80,6 (±33,2)	81,6 (±29,6)	**0,042**
Otros	59	33,0		81,2 (±24,0)	79,5 (±24,2)	82,5 (±19,9)	0,853		75,4 (±36,9)	75,8 (±35,6)	77,9 (±35,9)	0,764
**valor p** ^†^				0,293	0,861	0,465			0,537	0,446	0,922	
**5.º nivel: condiciones socioeconómicas, culturales y ambientales generales**												
**Renta familiar**												
< 3 salarios mínimos	107	59,8		78,8 (±22,3)	79,5 (±21,4)	79,7 (±20,4)	0,598		73,5 (±37,7)	80,3 (±32,5)	81,3 (±31,6)	**0,027**
> 3 salarios mínimos	72	40,2		82,2 ±20,9)	82,8 (±20,9)	85,0 (±17,6)	0,532		75,6 (±35,5)	77,0 (±36,2)	79,1 (±32,2)	0,856
**valor p** ^†^				0,311	0,299	0,142			0,843	0,711	0,510	

Leyenda: *Prueba de Friedman/

^§^Kruskal-Wallis/

^†^Mann-Whitney.

El análisis longitudinal de la asociación entre los DSS y los dominios del SF-36 reveló variaciones significativas a lo largo del segundo, cuarto y sexto mes de seguimiento. Estas diferencias se produjeron según las características sociodemográficas, los estilos de vida, las condiciones ambientales y las redes sociales (Tablas 1, 2 y 3).

**Tabla 2:** Asociación de los determinantes sociales de la salud de las personas con COVID-19 con las medias de los dominios dolor, estado general de salud y vitalidad del instrumento SF-36, comparando el 2.º, 4.º y 6.º mes – Fortaleza, CE, Brasil, 2023.

Dominios SF-36	Dolor media (±DE)					Estado general de salud media (±DE)					Vitalidad media (±DE)			
	2.º mes	4.º mes	6.º mes	p*		2.º mes	4.º mes	6.º mes	p*		2.º mes	4.º mes	6.º mes	p*
**1.ª camada: edad, sexo y factores hereditarios**														
**Edad**														
20–39	72,5 (±23,1)	73,5 (±22,4)	74,4 (±22,4)	0,514		64,4 (±20,5)	65,5 (±20,6)	66,4 (±21,2)	0,507		53,7 (±18,9)	54,3 (±22,0)	56,4 (±19,9)	0,246
40–59	66,4 (±24,4)	70,5 (±23,2)	72,0 (±23,6)	**0,015**		64,0 (±17,3)	68,7 (±18,3)	66,1 (±19,2)	0,096		56,8 (±19,4)	60,0 (±19,4)	62,2 (±19,0)	**0,006**
60–75	60,3 (±18,3)	65,1 (±27,5)	53,0 (±11,0)	0,485		52,3 (±8,9)	54,7 (±10,9)	55,3 (±12,2)	0,678		52,2 (±12,0)	53,8 (±13,8)	56,6 (±13,2)	0,482
**valor p** ^§^	0,155	0,555	**0,016**			0,090	0,074	0,152			0,563	0,278	0,217	
**Sexo**														
Masculino	76,3 (±22,5)	75,8 (±22,4)	78,0 (±21,4)	0,555		68,2 (±17,6)	72,0 (±18,9)	71,1 (±18,3)	**0,045**		61,4 (±19,3)	64,3 (±20,2)	63,9 (±18,6)	0,161
Mujeres	65,6 (±23,2)	69,5 (±23,0)	68,8 (±23,1)	**0,035**		60,5 (±19,8)	61,7 (±19,2)	62,0 (±20,9)	0,839		49,8 (±16,9)	50,3 (±19,6)	54,3 (±19,1)	**0,009**
**valor p** ^†^	**0,003**	0,068	**0,009**			**0,013**	**<0,001**	**0,004**			**<0,001**	**<0,001**	**0,001**	
**Raza/Color**														
Blanca	64,1 (±26,2)	69,6 (±24,4)	70,9 (±24,5)	**0,036**		62,2 (±22,8)	64,2 (±23,2)	63,2 (±22,4)	0,064		53,7 (±21,1)	54,0 (±22,4)	53,5 (±20,5)	0,856
Negra	72,5 (±22,4)	65,5 (±24,0)	75,3 (±21,5)	0,220		62,2 (±16,3)	63,8 (±19,2)	62,2 (±12,7)	0,334		55,0 (±18,5)	52,1 (±23,6)	53,8 (±18,9)	0,776
Parda	72,8 (±21,6)	74,9 (±21,6)	73,0 (±22,1)	0,073		64,8 (±17,6)	67,4 (±17,6)	67,9 (±20,2)	**0,035**		55,0 (±17,6)	58,1 (±19,5)	61,8 (±18,3)	**<0,001**
**valor p** ^§^	0,131	0,199	0,825			0,771	0,603	0,161			0,925	0,402	**0,024**	
**2.ª capa – Estilo de vida de los individuos**														
**Actividad física/semana**														
De 0 a 3 días	68,2 (±23,3)	70,2 (±22,7)	71,7 (±23,0)	**0,048**		62,8 (±18,9)	64,6 (±19,3)	64,7 (±19,8)	0,215		53,3 (±19,0)	54,8 (±21,5)	57,6 (±19,7)	**0,015**
4 a 7 días	77,7 (±22,7)	80,1 (±22,3)	76,3 (±21,8)	0,530		67,4 (±20,7)	72,1 (±20,4)	70,1 (±21,9)	0,512		60,1 (±17,1)	61,4 (±18,0)	60,8 (±18,4)	0,539
**valor p** ^†^	**0,028**	**0,021**	0,312			0,257	0,059	0,159			0,050	0,125	0,402	
**Consumo de frutas y verduras/semana**														
De 0 a 3 días	69,9 (±23,0)	71,6 (±21,9)	72,1 (±22,8)	0,174		61,1 (±20,0)	64,4 (±19,8)	62,9 (±20,7)	0,215		54,2 (±18,2)	55,7 (±21,8)	58,2 (±19,7)	0,056
4 a 7 días	70,2 (±24,1)	72,6 (±24,0)	73,1 (±22,9)	0,208		66,5 (±18,1)	67,8 (±19,5)	68,8 (±19,5)	0,589		55,1 (±19,4)	56,4 (±20,1)	58,2 (±19,3)	0,150
**valor p** ^†^	0,831	0,610	0,699			0,084	0,278	0,069			0,754	0,871	0,853	
**Consumo de fritos y productos procesados/semana**														
De 0 a 3 días	70,4 (±23,4)	72,9 (±23,0)	73,4 (±22,2)	0,168		64,4 (±19,1)	67,2 (±18,5)	67,3 (±19,7)	0,235		55,1 (±18,4)	56,6 (±21,0)	59,7 (±19,0)	**0,006**
4 a 7 días	69,1 (±23,7)	69,8 (±22,6)	70,5 (±24,3)	0,184		61,7 (±19,7)	62,7 (±22,4)	61,3 (±21,4)	0,344		53,2 (±19,8)	54,5 (±21,0)	54,2 (±20,2)	0,779
**valor p** ^†^	0,754	0,278	0,557			0,548	0,539	0,097			0,500	0,397	0,139	
**Exposición al sol/semana**														
De 0 a 3 días	71,2 (±23,7)	73,1 (±22,6)	73,3 (±22,7)	0,168		63,1 (±19,1)	66,4 (±19,1)	65,8 (±20,2)	**0,041**		54,8 (±17,7)	57,4 (±21,0)	60,0 (±18,1)	**0,007**
4 a 7 días	68,1 (±23,2)	70,6 (±23,4)	71,6 (±22,9)	0,214		64,6 (±19,5)	65,4 (±20,7)	65,6 (±20,5)	0,983		54,4 (±20,5)	54,0 (±20,9)	55,6 (±21,2)	0,593
**valor p** ^†^	0,323	0,509	0,670			0,501	0,896	0,895			0,999	0,340	0,200	
**Dificultad para dormir**														
Sí	66,8 (±24,5)	69,4 (±23,1)	70,1 (±23,0)	**0,011**		60,4 (±18,5)	63,6 (±20,1)	64,2 (±20,6)	**0,039**		51,3 (±19,6)	53,7 (±21,5)	54,7 (±20,4)	0,127
No	74,0 (±21,7)	75,5 (±22,3)	75,6 (±22,2)	0,860		67,8 (±19,5)	68,9 (±18,9)	67,6 (±19,8)	0,279		58,8 (±16,9)	59,0 (±20,1)	62,6 (±17,3)	0,053
**valor p** ^†^	**0,045**	0,099	0,118			**0,010**	0,074	0,368			**0,008**	0,060	**0,010**	
**Tabaquismo**														
Sí	79,7 (±24,0)	67,5 (±24,9)	79,6 (±16,2)	0,228		69,8 (±20,1)	62,3 (±15,4)	63,3 (±26,0)	0,058		60,0 (±17,5)	61,8 (±17,5)	68,1 (±16,4)	**0,040**
No	69,6 (±23,4)	72,3 (±22,9)	72,3 (±22,0)	**0,014**		63,4 (±19,2)	66,2 (±19,9)	65,8 (±20,1)	0,326		54,4 (±18,8)	55,8 (±21,1)	57,8 (±19,5)	**0,035**
**valor p** ^†^	0,243	0,481	0,455			0,452	0,561	0,740			0,408	0,449	0,169	
**Alcoholismo**														
Sí	73,8 (±23,5)	72,6 (±22,7)	75,3 (±21,1)	0,618		66,2 (±19,5)	66,7 (±19,3)	69,1 (±18,2)	0,377		56,0 (±18,6)	55,6 (±21,5)	59,3 (±20,4)	0,374
No	67,0 (±23,1)	71,7 (±23,2)	70,4 (±23,9)	**0,008**		61,7 (±18,9)	65,4 (±20,0)	63,1 (±21,5)	0,091		53,5 (±18,9)	58,5 (±20,6)	57,4 (±18,7)	**0,012**
**valor p** ^†^	0,060	0,801	0,216			0,125	0,826	0,092			0,376	0,903	0,414	
**3.ª capa: redes sociales y comunitarias**														
**Número de personas que residen**														
¿Vives solo o con alguien?	63,0 (±22,6)	69,0 (±22,8)	72,5 (±24,9)	0,152		59,8 (±19,0)	63,4 (±20,0)	61,8 (±27,7)	0,401		53,3 (±16,0)	54,4 (±20,6)	65,0 (±22,0)	0,087
≥ 2	70,4 (±23,5)	72,3 (±22,8)	72,6 (±22,7)	0,091		63,9 (±19,3)	66,1 (±19,7)	65,9 (±19,9)	0,169		54,7 (±18,9)	56,2 (±21,1)	57,9 (±19,3)	**0,025**
**valor p** ^†^	0,335	0,705	0,973			0,335	0,501	0,756			0,804	0,796	0,255	
**Vive con personas del grupo de riesgo**														
Sí	70,1 (±22,5)	72,2 (±22,1)	73,1 (±22,3)	0,273		64,6 (±20,1)	65,4 (±19,5)	67,3 (±19,8)	0,131		55,0 (±18,4)	53,7 (±21,2)	58,8 (±19,2)	**0,026**
No	69,9 (±24,5)	72,0 (±23,8)	72,1 (±23,3)	0,115		62,7 (±18,4)	66,5 (±19,9)	64,1 (±20,8)	0,026		54,3 (±19,3)	58,4 (±20,7)	57,6 (±19,7)	**0,011**
**valor p** ^†^	0,913	0,984	0,781			0,461	0,648	0,359			0,729	0,271	0,791	
**4.ª capa – Condiciones de vida**														
**Suministro de agua**														
Red de distribución	70,1 (±23,2)	71,8 (±22,9)	72,6 (±22,9)	0,062		63,6 (±19,3)	66,1 (±19,8)	65,7 (±20,4)	0,109		54,8 (±18,9)	56,6 (±21,0)	58,7 (±19,5)	**0,009**
Otros	68,1 (±27,7)	76,7 (±23,2)	77,2 (±20,9)	0,422		65,2 (±19,5)	64,0 (±18,2)	66,6 (±18,8)	1,000		51,3 (±17,3)	48,6 (±20,5)	50,9 (±17,8)	0,773
**valor p** ^†^	0,887	0,450	0,966			0,675	0,711	0,873			0,613	0,233	0,204	
**Desagüe de alcantarillado**														
Red de alcantarillado	68,6 (±23,5)	71,6 (±23,0)	71,7 (±23,6)	**0,045**		63,6 (±19,2)	65,7 (±20,3)	65,6 (±20,1)	0,182		53,3 (±18,1)	53,7 (±19,8)	56,9 (±18,5)	**0,017**
Otros	72,9 (±23,2)	73,2 (±22,9)	74,5 (±21,0)	0,622		63,8 (±19,4)	66,7 (±18,5)	66,1 (±20,9)	0,640		57,3 (±20,0)	61,0 (±22,6)	61,1 (±21,1)	0,302
**valor p** ^†^	0,216	0,623	0,434			0,881	0,746	0,866			0,155	**0,023**	0,195	
**5.ª capa – Condiciones socioeconómicas, culturales y ambientales generales**														
**Renta familiar**														
< 3 salarios mínimos	70,8 (±23,6)	73,9 (±22,8)	73,4 (±23,4)	0,150		63,4 (±18,8)	65,1 (±19,5)	63,8 (±20,4)	0,468		54,8 (±19,6)	57,0 (±21,3)	57,7 (±20,1)	**0,048**
> 3 salarios mínimos	68,9 (±23,4)	69,4 (±23,0)	71,4 (±22,0)	0,109		64,0 (±20,1)	67,3 (±19,9)	68,5 (±20,0)	0,130		54,4 (±17,5)	54,7 (±20,5)	59,0 (±18,5)	**0,034**
**valor p** ^†^	0,593	0,197	0,455			0,664	0,433	0,123			0,972	0,473	0,656	

Leyenda: *Prueba de Friedman/

^§^Kruskal-Wallis/

^†^Mann-Whitney.

**Tabla 3:** Asociación de los determinantes sociales de la salud de las personas con COVID-19 con las medias de los dominios aspectos sociales, aspectos emocionales y salud mental del instrumento SF-36, comparando el 2.º, 4.º y 6.º mes – Fortaleza, CE, Brasil, 2023.

Dominios SF-36	Aspectos sociales media (± DE)					Aspectos emocionales media (± DE)					Salud mental media (± DE)			
	2.º mes	4.º mes	6.º mes	p*		2.º mes	4.º mes	6.º mes	p*		2.º mes	4.º mes	6.º mes	p*
**1.ª capa: edad, sexo y factores hereditarios**														
**Edad**														
20–39	70,5 (±24,7)	73,3 (±24,8)	72,5 (±24,4)	0,220		63,4 (±40,1)	71,6 (±37,1)	70,7 (±38,4)	**0,025**		60,3 (±18,6)	63,5 (±19,3)	65,5 (±20,3)	**0,013**
40–59	72,3 (±23,9)	77,0 (±25,4)	79,0 (±23,2)	**0,002**		76,7 (±37,0)	74,4 (±40,1)	75,5 (±38,9)	0,705		64,0 (±19,7)	65,2 (±21,5)	67,5 (±19,5)	0,204
60–75	63,8 (±20,1)	69,4 (±26,5)	79,1 (±22,5)	0,125		59,2 (±49,3)	62,9 (±48,4)	77,7 (±44,0)	0,368		53,3 (±12,6)	53,7 (±15,1)	60,4 (±15,9)	**0,042**
**valor p** ^§^	0,508	0,479	0,212			0,056	0,596	0,537			0,163	0,240	0,482	
**Sexo**														
Masculino	77,5 (±22,5)	80,9 (±20,5)	81,9 (±21,4)	**0,014**		76,5 (±34,7)	82,8 (±30,8)	83,7 (±31,8)	0,179		68,3 (±17,6)	70,9 (±19,5)	72,3 (±18,5)	0,101
Mujeres	65,9 (±24,3)	69,6 (±26,9)	70,0 (±24,7)	0,061		60,9 (±42,2)	64,4 (±41,6)	64,7 (±41,3)	0,514		56,1 (±12,6)	58,4 (±18,6)	61,3 (±19,4)	**0,009**
**valor p** ^†^	**0,001**	**0,006**	**0,002**			**0,009**	**0,003**	**0,001**			**<0,001**	**<0,001**	**<0,001**	
**Raza/Color**														
Blanca	65,1 (±28,5)	71,9 (±26,5)	72,1 (±23,9)	**0,004**		64,9 (±41,0)	69,5 (±36,3)	71,3 (±38,0)	0,686		57,6 (±20,2)	61,8 (±20,4)	62,8 (±21,0)	**0,031**
Negra	72,0 (±26,4)	69,0 (±26,6)	72,0 (±28,4)	0,932		71,4 (±39,8)	61,9 (±43,8)	66,6 (±44,7)	0,907		62,8 (±20,0)	63,0 (±20,6)	64,1 (±18,8)	0,914
Parda	73,6 (±20,6)	76,7 (±23,7)	77,1 (±23,1)	0,065		67,9 (±39,6)	75,5 (±38,5)	74,5 (±38,0)	0,067		62,8 (±17,5)	64,7 (±19,6)	67,9 (±19,3)	**0,003**
**valor p** ^§^	0,283	0,340	0,423			0,707	0,144	0,781			0,317	0,662	0,354	
**2.ª capa – Estilo de vida de los individuos**														
**Actividad física/semana**														
0 a 3 días	70,5 (±24,8)	73,1 (±25,1)	75,0 (±24,4)	**0,015**		66,2 (±40,2)	71,9 (±38,6)	73,7 (±38,1)	0,036		60,6 (±19,8)	62,8 (±20,6)	65,6 (±19,9)	**0,003**
4 a 7 días	71,6 (±22,0)	79,4 (±24,4)	74,6 (±22,9)	**0,016**		72,5 (±38,9)	72,5 (±38,8)	67,6 (±41,4)	0,617		63,2 (±13,7)	66,9 (±16,5)	66,8 (±19,7)	0,417
**valor p** ^†^	0,952	0,144	0,844			0,328	0,957	0,415			0,452	0,249	0,721	
**Consumo de frutas y verduras/semana**														
De 0 a 3 días	73,2 (±23,7)	75,5 (±23,3)	76,7 (±23,3)	0,062		70,2 (±38,3)	71,6 (±38,4)	77,3 (±34,9)	0,094		60,7 (±19,2)	63,5 (±20,7)	66,0 (±19,7)	**0,014**
4 a 7 días	67,9 (±24,6)	72,9 (±26,9)	72,9 (±24,9)	**0,018**		64,3 (±41,7)	72,5 (±38,8)	76,4 (±42,0)	0,118		61,6 (±18,4)	63,6 (±19,1)	65,7 (±20,0)	0,075
**valor p** ^†^	0,131	0,658	0,299			0,443	0,800	0,152			0,731	0,964	0,986	
**Consumo de fritos y productos procesados/semana**														
De 0 a 3 días	71,4 (±23,6)	75,4 (±24,2)	77,3 (±23,0)	**<0,001**		67,6 (±39,8)	71,2 (±38,7)	75,8 (±37,4)	0,073		61,3 (±17,9)	63,7 (±20,0)	66,7 (±19,5)	**0,001**
4 a 7 días	68,7 (±25,9)	71,0 (±27,1)	68,2 (±25,7)	0,239		66,6 (±40,6)	74,3 (±38,4)	63,8 (±41,1)	**0,032**		60,6 (±21,1)	63,2 (±19,7)	63,5 (±20,6)	0,340
**valor p** ^†^	0,582	0,573	**0,025**			0,870	0,573	0,057			0,900	0,860	0,423	
**Exposición al sol/semana**														
0 a 3 días	72,9 (±23,1)	77,2 (±23,3)	78,4 (±23,1)	**0,003**		71,5 (±37,6)	75,8 (±37,6)	77,6 (±34,8)	0,180		61,1 (±18,4)	63,9 (±20,1)	68,0 (±18,4)	**0,002**
4 a 7 días	67,3 (±25,7)	69,6 (±26,9)	69,4 (±24,6)	0,212		60,9 (±42,8)	66,1 (±39,5)	64,7 (±43,1)	0,531		61,1 (±19,5)	63,0 (±19,6)	62,6 (±21,5)	0,312
**valor p** ^†^	0,164	0,061	**0,010**			0,134	0,050	0,069			0,841	0,798	0,140	
**Dificultad para dormir**														
Sí	65,0 (±25,5)	69,9 (±25,3)	71,3 (±24,4)	**0,004**		59,5 (±41,8)	63,9 (±41,6)	66,3 (±42,4)	0,290		55,5 (±19,0)	59,4 (±20,7)	62,1 (±20,9)	**<0,001**
No	77,8 (±20,5)	79,6 (±23,8)	79,3 (±23,0)	0,239		77,0 (±35,4)	82,0 (±31,7)	80,4 (±32,1)	0,345		68,1 (±16,0)	68,7 (±17,7)	70,6 (±17,4)	0,575
**valor p** ^†^	**0,001**	**0,006**	**0,016**			**0,004**	**0,002**	**0,038**			**<0,001**	**0,002**	**0,005**	
**Tabaquismo**														
Sí	78,1 (±19,7)	76,5 (±24,4)	79,6 (±25,8)	0,846		91,6 (±15,4)	87,5 (±17,2)	87,5 (±24,8)	0,819		61,5 (±11,6)	64,5 (±20,1)	67,5 (±18,3)	0,670
No	70,3 (±24,4)	74,1 (±25,1)	74,7 (±24,0)	**0,001**		66,2 (±40,4)	71,3 (±39,1)	71,9 (±39,1)	0,082		61,1 (±19,1)	63,5 (±19,9)	65,8 (±19,9)	**0,002**
**valor p** ^†^	0,449	0,808	0,533			0,104	0,455	0,313			0,964	0,958	0,911	
**Alcoholismo**														
Sí	73,8 (±23,2)	75,0 (±25,7)	78,3 (±23,4)	0,117		68,7 (±39,7)	75,5 (±37,6)	77,2 (±36,8)	0,069		61,8 (±16,7)	63,8 (±18,8)	67,0 (±19,3)	0,096
No	68,2 (±24,8)	73,7 (±24,5)	72,2 (±24,3)	**0,005**		66,3 (±40,3)	69,3 (±39,2)	69,0 (±39,9)	0,707		60,6 (±20,3)	63,4 (±20,8)	65,0 (±20,2)	**0,011**
**valor p** ^†^	0,129	0,639	0,090			0,702	0,261	0,156			0,836	0,899	0,521	
**3.ª capa: redes sociales y comunitarias**														
**Número de personas que residen**														
Vive sola o con una persona	79,1 (±15,3)	61,1 (±26,1)	72,2 (±23,1)	0,355		59,2 (±49,3)	44,4 (±44,0)	85,1 (±29,3)	0,115		58,2 (±19,7)	62,6 (±23,1)	71,5 (±19,1)	**0,008**
≥ 2	70,2 (±24,5)	75,0 (±24,8)	75,0 (±24,1)	**<0,001**		67,8 (±39,5)	73,5 (±37,8)	71,9 (±39,1)	0,116		61,3 (±18,8)	63,6 (±19,8)	65,6 (±19,8)	**0,008**
**valor p** ^†^	0,391	0,111	0,597			0,718	**0,048**	0,288			0,506	0,869	0,391	
**Vive con personas del grupo de riesgo**														
Sí	70,8 (±24,2)	75,4 (±24,2)	76,3 (±22,6)	**0,005**		67,4 (±40,8)	71,8 (±38,0)	74,4 (±39,3)	0,160		60,5 (±17,6)	61,1 (±19,1)	64,7 (±18,4)	0,131
No	70,6 (±24,4)	73,1 (±25,9)	73,4 (±25,4)	0,158		67,4 (±39,2)	72,2 (±39,3)	70,7 (±38,2)	0,397		61,7 (±19,9)	66,1 (±20,5)	67,1 (±21,2)	**0,004**
**valor p** ^†^	0,973	0,698	0,608			0,898	0,761	0,394			0,742	0,132	0,295	
**4.ª capa – Condiciones de vida**														
**Suministro de agua**														
Red de distribución	70,6 (±24,4)	74,5 (±25,2)	75,6 (±23,9)	**<0,001**		67,6 (±39,8)	72,8 (±38,1)	74,2 (±38,0)	0,081		61,1 (±19,0)	64,1 (±19,8)	66,2 (±19,9)	**<0,001**
Otros	71,5 (±21,7)	70,4 (±23,2)	63,6 (±24,6)	0,200		63,6 (±43,3)	60,6 (±44,2)	48,4 (±43,1)	0,174		61,8 (±15,4)	54,9 (±19,1)	60,3 (±17,9)	0,417
**valor p** ^†^	0,976	0,445	0,083			0,672	0,315	**0,020**			0,969	0,174	0,282	
**Desagüe**														
Red de alcantarillado	69,4 (±24,0)	73,1 (±24,7)	75,2 (±23,2)	**0,003**		65,8 (±39,4)	72,2 (±38,7)	73,0 (±37,2)	0,103		59,8 (±18,6)	61,4 (±19,1)	65,4 (±19,0)	**0,002**
Otros	73,3 (±24,6)	76,6 (±25,6)	74,3 (±25,8)	0,190		70,6 (±41,0)	71,7 (±38,5)	71,7 (±41,8)	0,837		63,7 (±19,0)	68,0 (±20,9)	66,7 (±21,5)	0,100
**valor p** ^†^	0,269	0,241	0,995			0,239	0,766	0,910			0,221	**0,029**	0,652	
**5.ª Capa – Condiciones socioeconómicas, culturales y ambientales generales**														
**Renta familiar**														
< 3 salarios mínimos	70,5 (±24,4)	74,2 (±25,2)	73,9 (±25,1)	**0,007**		69,1 (±39,3)	72,8 (±37,7)	73,2 (±38,9)	0,293		61,2 (±19,8)	64,1 (±20,4)	65,3 (±21,0)	**0,040**
> 3 salarios mínimos	71,0 (±24,1)	74,3 (±24,9)	76,3 (±22,4)	0,117		64,8 (±41,0)	70,8 (±39,9)	71,7 (±38,6)	0,404		61,0 (±17,2)	62,7 (±19,1)	66,7 (±17,9)	**0,025**
**valor p** ^†^	0,918	0,939	0,718			0,551	0,818	0,637			0,984	0,637	0,776	

Leyenda: *Prueba de Friedman/

^§^Kruskal-Wallis/

^†^Mann-Whitney.

En el primer grupo, los participantes de entre 40 y 59 años experimentaron una mejora significativa entre el segundo y el sexto mes en cuanto a capacidad funcional (p = 0,033), dolor (p = 0,042), vitalidad (p = 0,009) y aspectos sociales (p = 0,012). En cambio, entre los adultos jóvenes (de 20 a 39 años) y los adultos mayores (de 60 a 75 años), la mejora se centró en la salud mental, con una mayor variación entre el segundo y el sexto mes (p = 0,009 y p = 0,045, respectivamente). En comparación, los participantes de 20 a 39 años mantuvieron puntuaciones de capacidad funcional superiores a las de los de 40 a 59 años y de 60 a 75 años en el segundo mes (p = 0,016 y p = 0,021, respectivamente), manteniendo esta diferencia con respecto a los mayores en el cuarto (p = 0,026) y el sexto mes (p = 0,013). En el sexto mes, la edad también influyó en el dolor, siendo menor la percepción del dolor (puntuaciones más altas) en los jóvenes y las personas de mediana edad en comparación con los mayores (p = 0,004 y p = 0,010, respectivamente) (Tablas 1, 2 y 3).

En cuanto al sexo, los hombres presentaron una mejor recuperación en el estado general de salud (2.º y 4.º mes, p = 0,029) y en los aspectos sociales (2.º y 6.º mes, p = 0,040). Las mujeres mejoraron en cuanto al dolor (2.º y 4.º mes, p = 0,038), la vitalidad (4.º y 6.º mes, p = 0,035) y la salud mental (2.º y 6.º mes, p = 0,005). En la comparación entre grupos, los hombres presentaron medias superiores en prácticamente todos los ámbitos. Las diferencias significativas se mantuvieron a lo largo de los tres meses en cuanto a capacidad funcional, vitalidad y salud mental (p < 0,001 en todos los casos). En cuanto a las limitaciones por aspectos físicos, dolor, estado general de salud, aspectos sociales y aspectos emocionales, la superioridad masculina fue estadísticamente significativa en la mayoría de los puntos evaluados, con valores de p < 0,05 (Tablas 1, 2 y 3).

La raza/color influyó en el tiempo de recuperación: los blancos mejoraron los aspectos sociales y la salud mental de forma precoz (en el segundo y cuarto mes, p = 0,025 y p = 0,035, respectivamente) y tardía (en el cuarto y sexto mes en cuanto a los aspectos sociales, p = 0,011). Por su parte, las personas de raza mestiza concentraron su evolución entre el segundo y el sexto mes en el estado general de salud (p = 0,022) y la salud mental (p = 0,002). En el sexto mes, las personas de raza mestiza superaron a las de raza blanca en el ámbito de la vitalidad (p = 0,014) (Tablas 2 y 3).

En el segundo nivel, la frecuencia de la actividad física influyó en el patrón de mejora. Los sedentarios o poco activos (0 a 3 días/semana) evolucionaron entre el segundo y el sexto mes en cuanto a limitaciones en aspectos físicos (p = 0,019), vitalidad (p = 0,010), aspectos sociales (p = 0,022) y salud mental (p = 0,002). Por su parte, los más activos (de 4 a 7 días) presentaron una mejora puntual en los aspectos sociales (2.º y 4.º mes, p = 0,018), pero mantuvieron medias consistentemente superiores a las de los poco activos en capacidad funcional en el 2.º (p = 0,010), 4.º (p = 0,010) y 6.º mes (p = 0,042); limitaciones por aspectos físicos en el segundo mes (p = 0,009); y dolor en el segundo (p = 0,028) y cuarto mes (p = 0,021) (Tablas 1, 2 y 3).

La alimentación y la exposición al sol también influyeron en la evolución. Un bajo consumo de frutas y verduras (de 0 a 3 días a la semana) se asoció a una mejora de la salud mental, especialmente entre el segundo y el sexto mes (p = 0,008). Por su parte, entre quienes consumían con mayor frecuencia (de 4 a 7 días), se observó una mejora en los aspectos sociales con una diferencia significativa entre el segundo y el cuarto mes (p = 0,046). Un menor consumo de alimentos fritos y procesados se asoció a una mejora entre el segundo y el sexto mes en la capacidad funcional (p = 0,026), la vitalidad (p = 0,005) y la salud mental (p < 0,001). El dominio de los aspectos sociales presentó diferencias entre el segundo y el cuarto mes (p = 0,036). Aquellos que consumían alimentos fritos y procesados de 0 a 3 días a la semana presentaron mejores puntuaciones en el dominio de los aspectos sociales en el sexto mes (p = 0,025) (Tablas 1, 2 y 3).

Los participantes con menor exposición solar (de 0 a 3 días a la semana) mostraron mejoras observadas entre el segundo y el sexto mes en cuanto a vitalidad (p = 0,005), aspectos sociales (p = 0,010) y salud mental (p = 0,001), y entre el segundo y el cuarto mes en cuanto al estado general de salud (p = 0,030) y los aspectos sociales (p = 0,046). Aquellos que se exponían al sol de 0 a 3 días a la semana también presentaron medias más altas en los aspectos sociales en el sexto mes (p = 0,010) (Tablas 2 y 3).

La dificultad para dormir presentó diferencias entre el segundo y el cuarto mes en los ámbitos del dolor (p = 0,036), los aspectos sociales (p = 0,009) y la salud mental (p < 0,001), además de una diferencia entre el segundo y el sexto mes en cuanto al estado general de salud (p = 0,030). Entre los no fumadores, se observó una diferencia en los siguientes ámbitos: dolor (2.º y 4.º mes, p = 0,029); vitalidad (2.º y 6.º mes, p = 0,022); aspectos sociales (2.º y 4.º mes, p = 0,014, y 4.º y 6.º mes, p = 0,008); y salud mental (2.º y 6.º mes, p = 0,001) (Tablas 2 y 3).

Las personas que no consumían alcohol mostraron una evolución positiva en cuanto al dolor, con diferencias significativas entre el segundo y el cuarto mes en los ámbitos del dolor (p = 0,012) y los aspectos sociales (p = 0,012). Entre el segundo y el sexto mes, las diferencias se observaron en los dominios de vitalidad (p = 0,010), aspectos sociales (p = 0,031) y salud mental (p = 0,006) (tablas 2 y 3).

En el tercer nivel, el apoyo social fue relevante. Vivir solo o con una persona presentó una mejora significativa únicamente en la salud mental (meses 2 y 6, p = 0,007). Aquellos que vivían con dos o más personas mostraron mejoras en la vitalidad (meses 2 y 6, p = 0,016) y la salud mental (meses 2 y 6, p = 0,005). En el ámbito de los aspectos sociales, la diferencia se produjo entre el 2.º y el 4.º mes (p = 0,005) (tablas 2 y 3).

La convivencia con personas del grupo de riesgo se asoció a mejoras entre el cuarto y el sexto mes en cuanto a la vitalidad (p = 0,021) y entre el segundo y el cuarto mes en cuanto a los aspectos sociales (p = 0,031). Entre quienes no convivían con personas del grupo de riesgo, se observaron diferencias estadísticamente significativas entre el segundo y el cuarto mes en los ámbitos de vitalidad (p = 0,013) y salud mental (p = 0,033), así como entre el segundo y el cuarto mes (p = 0,039) y entre el cuarto y el sexto mes (p = 0,036) en el dominio estado general de salud (Tablas 2 y 3).

En el cuarto nivel, “condiciones de vida”, los participantes que residían en lugares con suministro de agua de la red pública mostraron diferencias significativas en los ámbitos de “vitalidad” entre el segundo y el sexto mes (p = 0,006), “aspectos sociales” entre el segundo y el cuarto mes (p = 0,010) y de salud mental entre el segundo y el cuarto mes (p = 0,022). Aquellos que disponían de suministro de agua de la red pública mostraron mejores medias en el ámbito de los aspectos emocionales en el sexto mes (p = 0,020) (Tablas 2 y 3).

En cuanto al tipo de saneamiento, las personas residentes en zonas con red de alcantarillado presentaron mejoras significativas en vitalidad entre el segundo y el sexto mes (p = 0,012), en aspectos sociales entre el segundo y el cuarto mes (p = 0,045) y en salud mental entre el cuarto y el sexto mes (p = 0,033) (Tablas 2 y 3).

En el quinto nivel, los participantes con unos ingresos familiares mensuales inferiores a tres salarios mínimos mostraron una mejora con diferencias significativas en los aspectos sociales (en el segundo y cuarto mes, p = 0,022) y de salud mental (en el segundo y sexto mes, p = 0,022). En el caso de los participantes con ingresos superiores a tres salarios mínimos, las diferencias se observaron en vitalidad (4.º y 6.º mes, p = 0,041) y salud mental (2.º y 6.º mes, p = 0,012) (Tablas 2 y 3).

## DISCUSIÓN

Los resultados ponen de manifiesto que los DSS influyen en la calidad de vida de los pacientes diagnosticados con COVID-19 a lo largo del tiempo, lo que confirma que la trayectoria de mejoría no depende únicamente de la resolución clínica, sino también de las condiciones estructurales y conductuales. El predominio de adultos jóvenes concuerda con el cambio en el perfil epidemiológico de la COVID-19^(15)^, en contraste con la edad promedio más elevada observada al inicio de la pandemia^(16)^, lo que puede haber favorecido la recuperación funcional observada, en contraste con la peor CV reportada en personas mayores tras la infección^(17)^.

En cuanto al sexo, la mayor proporción de mujeres refleja la participación femenina en las prácticas de cuidado de la salud y las pruebas de COVID-19^(18)^. Sin embargo, los hombres presentaron una mejor recuperación en aspectos físicos y sociales, mientras que las mujeres obtuvieron mejores resultados en salud mental. Esta disparidad sugiere diferencias en la forma de afrontar la pandemia y en su impacto psicosocial entre los géneros^(19)^.

En cuanto a la raza/color, el predominio de personas que se autodefinen como mestizas refleja la composición demográfica brasileña^(20)^ y los hallazgos de estudios anteriores con poblaciones afectadas por la COVID-19^(20,21,22)^. La distinción en la recuperación entre blancos (mejores en salud mental) y pardos (aspectos físicos) refuerza el peso de las desigualdades estructurales y del estrés social que enfrentan los grupos racializados en Brasil^(22)^.

El estilo de vida se reveló como un factor central. El alto nivel de sedentarismo refleja los impactos del aislamiento social durante la pandemia^(23)^, pero la práctica regular de actividad física se confirmó como un factor protector para la vitalidad y la salud mental^(24)^. Se observaron patrones similares en la alimentación y el sueño: el consumo de alimentos saludables se asoció con mejores resultados psicosociales^(25)^, mientras que los trastornos del sueño y la baja exposición al sol se asociaron con peores indicadores en diversos ámbitos, lo que evidencia la importancia de la luz solar para la síntesis de vitamina D y el equilibrio emocional, y el impacto del confinamiento en el ritmo circadiano y el bienestar^(26)^. Estos hallazgos refuerzan el papel de los comportamientos relacionados con el estilo de vida, el nivel intermedio del modelo de Dahlgren y Whitehead, en la modulación de la calidad de vida durante el período pos-COVID-19.

El tabaquismo presentó una baja prevalencia, lo que corrobora los estudios que señalan una mayor preocupación por las enfermedades respiratorias durante la crisis sanitaria^(27)^. La ausencia de tabaquismo y el no consumo de alcohol actuaron como factores de protección para la calidad de vida general, contrariamente a las tendencias de aumento del consumo durante la pandemia^(23,28)^.

En el ámbito de las redes sociales y comunitarias, la convivencia con un mayor número de personas o grupos de riesgo tuvo un impacto negativo en la vitalidad y el dolor, lo que refleja la dificultad del aislamiento y la sobrecarga de cuidados en los entornos^(20,29)^.

En cuanto a las condiciones de vivienda, el acceso a servicios básicos de saneamiento (agua y alcantarillado) resultó fundamental para la recuperación en casi todos los dominios de la CV^(30)^. El ingreso familiar, en su mayoría bajo, revela un importante marcador de vulnerabilidad que puede aumentar el riesgo de exposición y dificultar el acceso a recursos esenciales^(31)^. Sin embargo, este estudio reveló que las familias con ingresos de hasta tres salarios mínimos presentaron mejores resultados en los aspectos físicos y sociales, mientras que las de ingresos más altos destacaron únicamente en el dominio de la vitalidad. Este matiz sugiere que los ingresos, aunque son un DSS crítico, pueden tener interacciones complejas con otros factores de protección o vulnerabilidad pos-COVID-19.

La interpretación de los hallazgos a la luz del modelo de Dahlgren y Whitehead^(6)^ permite comprender que los DSS actúan de manera interdependiente en la recuperación de la CV tras la COVID-19^(32)^. Factores individuales, como la edad, el sexo y la raza/color, interactúan con comportamientos relacionados con el estilo de vida, las redes sociales y comunitarias, así como con las condiciones de vivienda y los ingresos, influyendo de manera distinta en los dominios físico y mental de la CV a lo largo del seguimiento. En este sentido, se observó que el estilo de vida, las condiciones de vivienda, los ingresos y las redes de apoyo modularon la trayectoria de recuperación, lo que evidencia que las personas expuestas a contextos sociales más favorables presentaron una mejor evolución en determinados dominios del SF-36. Estos hallazgos refuerzan que la recuperación pos-COVID-19 no ocurre de manera homogénea, sino que está permeada por desigualdades sociales que influyen en el proceso de rehabilitación.

La evolución positiva de las puntuaciones del SF-36 a lo largo de los seis meses es consistente con estudios internacionales^(14,33)^, lo que indica una restauración progresiva de la salud. El seguimiento durante un período de seis meses fue esencial para determinar los impactos de los DSS a lo largo del tiempo, así como para observar la tendencia de evolución de la CV de las personas afectadas por la COVID-19. La influencia de los DSS refuerza la necesidad de estrategias intersectoriales que trabajen con un enfoque dirigido principalmente a los DSS que ejercen impactos directos en la CV de las personas. La enfermería, en la atención primaria, debe liderar la planificación de cuidados que integren la promoción de hábitos saludables y el fortalecimiento de redes de apoyo, considerando las desigualdades regionales para garantizar una rehabilitación equitativa^(12)^.

Es importante reconocer las limitaciones de este estudio. La restricción geográfica a los estados de Ceará y Maranhão limita la generalización de los hallazgos a nivel nacional. Además, no se controlaron posibles factores de interferencia a lo largo del seguimiento, como la reinfección por COVID-19, el agravamiento del cuadro clínico, el acceso a servicios de rehabilitación o los cambios en las condiciones de vida de los participantes, que podrían influir en las puntuaciones de calidad de vida observadas. La deserción de participantes a lo largo del seguimiento (de 391 a 179 participantes), aunque prevista en el cálculo de la muestra, puede haber atenuado la fuerza de algunas asociaciones. A pesar de ello, los hallazgos ofrecen información relevante para la gestión pública y la práctica clínica en el contexto pospandémico.

## CONCLUSIÓN

Se concluye que la calidad de vida de las personas en la fase pos-COVID-19 presentó una mejora progresiva y estadísticamente significativa a lo largo del tiempo, y que esta trayectoria se vio intrínsecamente influenciada por los DSS. Los factores relacionados con el estilo de vida, las condiciones de vivienda, los ingresos y las redes de apoyo modularon la recuperación de los dominios físico y mental, lo que evidencia que el proceso de rehabilitación no ocurre de manera homogénea, sino que está impregnado de desigualdades sociales que impactan la evolución de la CV.

Estos hallazgos refuerzan la necesidad de que la enfermería y la gestión pública implementen estrategias longitudinales que trasciendan el manejo clínico de la condición pos-COVID-19. En este sentido, se destacan las acciones intersectoriales orientadas a la promoción de hábitos saludables, el incentivo a la práctica de actividad física, la educación en salud sobre alimentación y sueño, el fortalecimiento de las redes de apoyo social, así como las políticas públicas orientadas a la mejora de las condiciones de vivienda, el saneamiento básico y el acceso equitativo a los servicios de salud y rehabilitación.

Como contribución científica, este estudio avanza al analizar longitudinalmente la influencia de los DSS en la CV post-COVID-19, evidenciando cómo las diferentes capas del modelo de Dahlgren y Whitehead interactúan en el proceso de recuperación. Estos resultados contribuyen a ampliar la comprensión de las desigualdades en el contexto pospandémico y ofrecen información para la planificación de intervenciones de enfermería en la atención primaria de salud, orientadas a la equidad y la promoción de la salud. Se recomienda replicar el estudio en otros contextos para ampliar la comprensión del impacto de la pandemia en la población brasileña y respaldar políticas de salud orientadas a la equidad.

## Data Availability

Todo el conjunto de datos que apoya los resultados de este estudio fue publicado en el propio artículo.
